# Genetic structure of *Rattus rattus* populations in an endemic plague focus in Madagascar: Implications for rodent surveillance and management

**DOI:** 10.1371/journal.pone.0334819

**Published:** 2026-07-13

**Authors:** Mamionah N. J. Parany, Anne Loiseau, Philippe Gauthier, Soanandrasana Rahelinirina, Gauthier Dobigny, Olivier Gorgé, Eric Valade, Minoarisoa Rajerison, Beza Ramasindrazana, Carine Brouat

**Affiliations:** 1 Institut Pasteur de Madagascar, Plague Unit, Antananarivo, Madagascar; 2 University of Antananarivo, Faculty of Science, Antananarivo, Madagascar; 3 CBGP, IRD, CIRAD, INRAE, Institut Agro, Université de Montpellier, Montpellier, France; 4 Institut de Recherche Biomédicales des Armées, Brétigny-sur-Orge, France; National Cheng Kung University, TAIWAN

## Abstract

**Background:**

Plague remains a major public health concern in Madagascar. In the Central Highlands, where the disease is still endemic, the black rat (*Rattus rattus*) is the main reservoir of the causative agent *Yersinia pestis*. Understanding its population dynamics and structure is therefore crucial to inform control strategies, as dispersal may greatly limit the effectiveness of local interventions during outbreaks. In this context, our study investigates the genetic structure of *R. rattus* populations at a fine geographical scale and across two different years.

**Methodology/Principal findings:**

Sampling was conducted in six villages of the Azobenzene district, both inside houses and in habitat outside villages. A total of 480 individuals, captured in March – May 2019 and 2020, were genotyped at 18 microsatellite loci. Our results showed that genetic diversity levels were relatively similar among villages and years. However, subpopulations living outside villages displayed significantly higher genetic diversity and lower genetic differentiation levels than those from inside houses, indicating larger effective population sizes outside villages in the cultivated habitats. These findings suggest more restricted movement among rat subpopulations from the houses, and greater connectivity among subpopulations living outside villages. However, overall genetic differentiation was rather low, suggesting extensive dispersal of rats at the scale of the district, facilitating rapid recolonization after local control efforts.

**Conclusion:**

Because of the recolonization problem, an integrated approach combining flea control inside houses together with measures to reduce human-rodent contact would thus appear more appropriate than rodent control only to limit plague transmission.

## Introduction

Plague, though often perceived as a disease of the past, continues to pose serious public health challenges in certain regions of the world, particularly in Africa [1]. This zoonosis, caused by the bacterium *Yersinia pestis*, persists in natural foci. It affects a large variety of mammals, including rodents that act as reservoirs, while their fleas are vectors [2,3]. Because the pathogen circulates among many wild reservoir populations [4], plague cannot be eradicated [1]. Human outbreaks typically occur within or near these natural foci, and their occurrence is partly related to the spatio-temporal dynamics of the reservoirs and vectors, and notably of commensal rodents and their fleas living in close contact with humans [5–7]. Understanding these dynamics is therefore critical for improving surveillance and anticipating epidemic outbreaks on the one hand, and for implementing early effective control strategies on the other hand [8].

Madagascar is one of the major plague foci, with up to 80% of human plague cases reported worldwide annually [9]. The disease was introduced on the island over a century ago via maritime transports [10], and it rapidly moved to and became endemic in the Central Highlands, typically at elevation above 800 m [11]. In rural areas of the highlands, the black rat (*Rattus rattus*) is the main reservoir of *Y. pestis*, and occurs in villages and their surrounding environments [11]*.* Habitats inside houses and outside villages constitute distinct ecological contexts for rat populations, characterized by contrasting demographic dynamics: reproduction is continuous and relatively stable inside houses, whereas marked seasonal fluctuations occur outdoors [12]. The habitats are also associated with distinct flea assemblages [6,13]: *Xenopsylla cheopis*, a cosmopolitan flea, is found inside houses, whereas *Synopsyllus fonquerniei,* an endemic species, is mainly found outdoors. Rats sampled outdoors exhibit higher plague seroprevalence than those captured inside houses, suggesting potential differences in the transmission dynamics of *Y. pestis* [14].

Human plague cases are predominantly reported during the hot and humid season, generally from September to April, a period corresponding to a low abundance of *R. rattus* populations outdoors [12], while coinciding with a peak of abundance of *S. fonquerniei* [6]. During plague outbreaks, rodent and flea control are usually implemented locally, i.e., inside and around houses where human cases have been reported, in order to reduce the populations of reservoirs and vectors. However, the high reproductive potential of *R. rattus* (up to 2–3 generations of rats per year) and its substantial mobility between habitats [15,16] may limit the effectiveness of strictly local control strategies. Genetic data provide valuable insights into the spatio-temporal structure, population size and dispersal of reservoir populations, thereby informing rodent control strategies [17,18]. In Madagascar, previous studies based on microsatellite markers have documented significant genetic differentiation among habitats [19,20], and spatially variable levels of genetic structure across the Central Highlands, shaped by topography, which may be associated with the heterogeneous distribution of human cases [20]. However, little is known about the short-term temporal stability of genetic structure at a local scale, despite its importance for interpreting spatial patterns. Black rats have short generation times (approximately 4–6 months) and high reproductive rates [21,22]. Populations in the Central Highlands may be affected by plague-induced mortality and human-mediated dispersal among villages. These processes could result in rapid shifts in allele frequencies and local genetic composition, which may in turn affect spatial population structure. Because rats reproduce continuously inside houses, and experience seasonal dynamics outdoors, genetic structuring might be more detectable among subpopulations inside houses, which would be more stable across time and less affected by dispersal events, than among subpopulations sampled outside villages, which could experience greater spatial connectivity.

In this study, we investigated the population genetic structure of the main plague reservoir, *R. rattus*, within one district of the Central Highlands of Madagascar where plague is endemic and where human plague cases occur every year (Unpublished data, Central Laboratory for Plague, Institut Pasteur de Madagascar). We assessed genetic diversity and estimated effective population size (*N*_*e*_), and examined the short-term stability of genetic structuring between two consecutive years in relation to habitat type (inside houses vs. outside villages). By evaluating both spatial and short-term temporal patterns, we aimed to determine whether observed genetic differentiation reflects stable local populations or transient demographic dynamics shaped by recurrent turnover and recolonization, and to identify the spatial scale most relevant for implementing rodent control measures.

## Materials and methods

### Study areas and small mammals trapping

**Fig 1:**
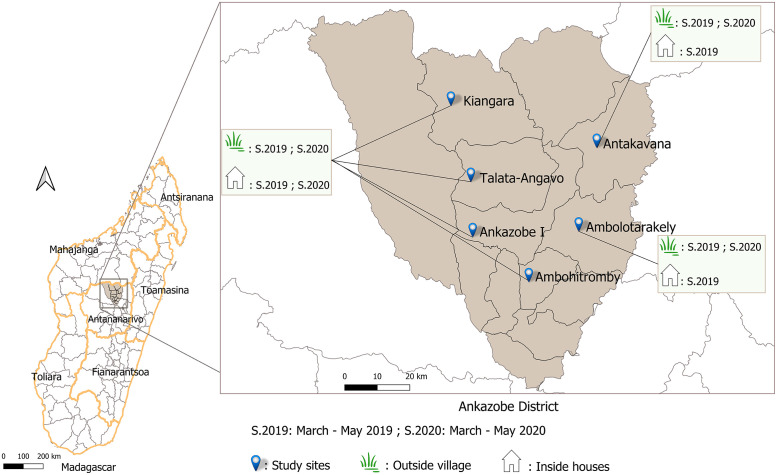
Sampling localities in Ankazobe district. Trapping was performed in two habitat types (i.e., inside houses and outside villages) during six sessions between 2018 and 2020, but only two sessions (March-May 2019 and March-May 2020) were considered in genetic analyses.

This study was carried out in Ankazobe district, covering a total area of 7,458 km^2^, and located in the Central Highlands (mean elevation of 1,190 m) of Madagascar. Six localities were investigated, namely Ambohitromby (18°25’S/ 47°9’E), Ambolotarakely (18°1’S/ 47°23’E), Antakavana (18°1’S/ 47°23’E), Kiangara (17°54’S/ 47°1’E), Talata-Angavo (18°12’S/ 47°5’E) and Ankazobe I (18°12’S/ 47°5’E) (Fig 1). In these localities, villages are typically built on the top of hills, composed of 20–150 households and dwellings are mostly made of mud or brick with thatched roofs. Hill slopes are characterized by grassy areas while the lowlands (50–1,000 m away from the houses) are covered by rice paddies and food crops (vegetables, cassava or maize). The distance between localities varies from 10–80 km (Fig 1). To our knowledge, no organized rat population control campaigns had been conducted at the scale of the studied villages prior to the initiation of this study.

Trapping was systematically performed in two habitat types (i.e., inside houses and outside villages) separated by distances ranging from 11 to 234 m (mean distance: 106 m) in each locality (Fig 1). A detailed description of field protocols was already provided in Parany et al*.* (2026) [12]. Six trapping sessions (TS) were carried out in the six localities, among which two were considered for genetic analyses. Both sessions were conducted during the same period for two successive years: sampling 1 in March – May 2019 (S.2019), and sampling 2 in March-May 2020 (S.2020) (Fig 1). In each village, one BTS (Besançon Technique Service, 30L × 10W × 10H cm, BTS Company©, Besançon, France) and one Sherman (23L × 7.5W × 9H cm, H.B. Sherman Traps Inc©., Tallahassee, Florida, USA) traps were set inside 18 houses for three successive nights. Outside each village, 84 BTS traps were placed along potential rodent pathways of reservoirs and/or near crop fields in a single long transect 830 m long with 10-m intervals between traps, and were set for three successive nights. All traps were georeferenced using GPS and baited every morning with a mixture of dried onions and dry fish, and checked twice a day. Traps that had captured rodents were replaced every morning.

### Data and sample collection

Each captured rodent was euthanized by cervical dislocation. Body measurements allowed identification of rodents at the species level, since terrestrial small mammals from the present experimental setting cannot suffer from potential taxonomic ambiguities [23]. Blood was collected by cardiac puncture and centrifuged to obtain sera samples for serological analyses. Furthermore, one kidney from each individual was sampled then stored in 95° ethanol for subsequent molecular investigation.

### Serological diagnosis of plague

Small mammal sera were tested for IgG antibodies specific to the *Y. pestis* F1 antigen using a well-established ELISA protocol [24]. Given the low number of seropositive rodents sampled during the two sessions considered for genetic analyses (see below), plague seroprevalence was then calculated as the percentage of seropositive individuals per locality, taking all individuals (including other species than *R. rattus*) sampled during the six sessions into account.

### Microsatellite genotyping

Molecular analyses were conducted on 11–40 *R. rattus* per habitat (i.e., inside houses or outside villages), locality and session (Table 1). For each individual, total DNA was extracted from kidney using QIAamp DNA Mini Kit (Qiagen, Hilden, Germany) as recommended by the manufacturer. Microsatellite loci amplification was carried out in multiplex using a panel of 18 microsatellite markers, eight of which (namely, D10Rat20, D11Mgh5, D11Rat56, D16Rat81, D2Mgh14, D7Rat13, D5Rat83 and D18Rat75) being originally developed for *R. norvegicus* [25] while ten others (namely, Rr14, Rr17, Rr21, Rr22, Rr54, Rr67, Rr68, Rr93, Rr107 and Rr114) being *R. rattus*-specific (See S1 Table for details of microsatellite markers) [26]. Polymerase Chain Reactions (PCR) and genotyping were conducted according to previously described procedures [27,28]. Genotyping was performed with an ABI 3130xl Genetic Analyzers (Applied Biosystems, Foster City, CA, USA) and microsatellite profiles were read, analysed and manually corrected in GeneMapper v.6 software.

**Table 1:** Genetic diversity estimates per subpopulation of *Rattus rattus,* and number of plague seropositive rodents and global plague seroprevalence levels for each locality.

Locality	Habitat	Session	N	*H*_E_	*r*	*F*_IS_	*Ne*	Nsero + / Nsampled (species)	SP
Ambolotarakely (ABK)	Outside villages	S.2019	22	0.72	6.3	0.06	1301 [141; ∞]	0/69	
		S.2020	19	0.72	6.1	0.03	132 [64; ∞]	0/91	
	Inside houses	S.2019	23	0.72	6	0.129	13 [11; 15]	0/23	
		S.2020	NA	NA	NA	NA	NA	0/16	
	Overall		**64**	**0.72**	**6.12**	**0.07**	**482 ± 546**		**0.21**
Ambohitromby (AMB)	Outside villages	S.2019	40	0.72	6.2	−0.007	370 [178; ∞]	0/42	
		S.2020	22	0.71	6	−0.016	33 [26; 44]	0/80	
	Inside houses	S.2019	22	0.72	5.7	0.086	22 [18; 28]	0/14	
		S.2020	11	0.65	5	0.106	6 [3; 8]	0/13	
	**Overall**		**95**	**0.7**	**5.72**	**0.04**	**107.75 ± 131.125**		**0.37**
Ankazobe I (ANK)	Outside villages	S.2019	22	0.69	5.8	0.052	50 [35; 84]	0/65	
		S.2020	21	0.7	5.8	0.106	42 [31; 64]	2 (*Rattus rattus*)/96	
	Inside houses	S.2019	22	0.68	5.2	−0.043	10 [9; 12]	0/17	
		S.2020	18	0.69	5.9	0.093	35 [25; 53]	0/23	
	**Overall**		**83**	**0.69**	**5.68**	**0.05**	**34.25 ± 12.125**		**0.41**
Antakavana (ANT)	Outside villages	S.2019	25	0.73	6.3	0.075	748 [156; ∞]	0/30	
		S.2020	21	0.73	6.3	0.076	147 [74; 1623]	0/34	
	Inside houses	S.2019	17	0.67	5.4	0.001	18 [14; 24]	0/14	
		S.2020	NA	NA	NA	NA	NA	0/10	
	**Overall**		**63**	**0.71**	**6**	**0.05**	**304.333 ± 295.778**		**0.71**
Kiangara (KIA)	Outside villages	S.2019	21	0.74	6.3	0.037	113 [61;542]	0/47	
		S.2020	22	0.72	6.2	0.037	49 [36; 73]	0/97	
	Inside houses	S.2019	17	0.7	5.2	0.073	11 [10; 13]	0/29	
		S.2020	22	0.69	5.5	−0.017	15 [11; 19]	1 (*Mus musculus*)/24	
	**Overall**		**82**	**0.71**	**5.79**	**0.03**	**47 ± 34**		**0.20**
Talata-Angavo (TLA)	Outside villages	S.2019	22	0.74	6.1	0.045	132 [69; 807]	0/52	
		S.2020	23	0.73	5.9	0.059	169 [80; ∞]	1 (*Rattus rattus*)/80	
	Inside houses	S.2019	22	0.72	6	0.066	27 [22; 34]	0/28	
		S.2020	22	0.69	5.5	0.163	20 [16; 25]	1 (*Mus musculus*)/11	
	**Overall**		**89**	**0.72**	**5.89**	**0.08**	**87 ± 63.500**		**0.63**

N: number of genotyped *R. rattus* individuals per subpopulations; *H*_*E*_: expected heterozygosity; *r*: allelic richness; *N*_*e*_: effective population size; NA: no estimate because of low sample size. Nsero + / Nsampled: number of seropositive (and species)/ number of sampled small mammals (including other species than *R. rattus*) captured; SP: plague seroprevalence in all small mammals sampled at the scale of the locality across all sessions.

### Data analyses

Individuals belonging to a given habitat (inside house or outside village), locality and trapping session (S.2019 or S.2020) were considered as a distinct subpopulation in the subsequent analyses. Deviations from Hardy-Weinberg equilibrium (HWE) within subpopulations and genotypic linkage disequilibrium (LD) between pairs of loci were assessed using GENEPOP v.4 [29]. We corrected for multiple testing by the false discovery rate approach [30] implemented in the QVALUE package of R v. 4.3.2 [31,32]. We used MICRO-CHECKER v.2.2.3 [33] to test whether heterozygote deficiencies could be accounted for by the existence of null alleles.

Genetic diversity of each subpopulation was estimated with FSTAT v.2.9.4 [34] using three estimators: 1) allelic richness (*r*) calculated for a minimum of 10 individuals using the rarefaction procedure, 2) Nei’s unbiased genetic diversity (*H*_*E*_, [35]) and 3) the inbreeding coefficient *F*_*IS*_. Effective population size (*N*_*e*_) was estimated for each subpopulation with the Linkage Disequilibrium (LD) methods using NE ESTIMATOR v.2.1 [36]. Alleles with a frequency ≥ 0.02 were used to minimise possible bias [37]. We compared *r*, *H*_*E*_, *F*_*IS*_, and relatedness (calculated with FSTAT [38]) between habitats and between sessions using FSTAT (10,000 permutations), and *N*_*e*_ using non parametric Kruskall-Wallis tests in R [32]. Very low numbers of seropositive rodents did not enable us to compare seroprevalence levels among habitats or sessions (see below). We thus only explored whether variation in plague seroprevalence among localities was associated with differences in genetic diversity and effective population size. Specifically, we assessed the relationships between mean *H*_*E,*_ *r* and *F*_*IS*_ on the one hand, and plague seroprevalence levels on the other hand, using Spearman’s rank non parametric tests in R [32].

Genetic differentiation among each pair of subpopulations from each locality was summarized by calculating pairwise *F*_*ST*_ estimates [39], with 95% confidence intervals (CIs) estimated by bootstrap resampling across loci using FSTAT (10,000 repetitions). *F*_*ST*_ values were compared among habitats and sessions with FSTAT (10,000 permutations).

Genetic structure was further examined in several ways. First, we assessed whether locality may explain population genetic structure by performing an analysis of molecular variance (AMOVA; [40]) with ARLEQUIN v.2.000 [41] using the locus-by-locus option. The variance components were tested using randomization (1,000 permutations). Second, we used G-based (log-likelihood ratio) randomization tests [42] to evaluate the effects of habitat (inside houses *vs*. outside villages) and sessions on genetic structure using FSTAT (10,000 permutations of individuals between subpopulations for each analysis). Independent tests of pairwise genetic differentiation were combined using the generalized binomial procedure implemented in Multitest v.1.2 [43].

Under a model of isolation by distance (IBD), genetic distance between subpopulations is expected to increase with geographic distance. IBD was investigated by regressing pairwise estimates of *F*_*ST*_/(1 – *F*_*ST*_) against the logarithm of the Euclidean geographic distances between subpopulations [44], calculated using GPS coordinates. Mantel tests were performed to test the correlation between matrices of genetic differentiation and geographic distance in GENEPOP (10,000 permutations) for the whole dataset as well as considering each habitat separately.

Genetic structure was also examined using the Bayesian clustering approach implemented in STRUCTURE v.2.3.4 [45] in order to estimate the number of homogeneous genetic groups (*K*) in our dataset. Analyses were performed with a model including admixture and correlated allele frequencies, for *K* ranging from 1 to10, using the LOCPRIOR option. Each run included 500,000 burn-in iterations following by 800,000 iterations. We performed 10 independent analyses for each *K* value. The number of genetic groups was inferred by the Delta *K* (ΔK) method applied to the log probabilities of data [46]. We also used Discriminant Principal Component Analysis (DAPC; which can handle the absence of HW equilibrium) under R using *adegenet* and *devtools* packages, as a confirmatory analysis [47]. We determined the genetic groups (*K*) with the Bayesian Information Criterion (BIC), using the delta-BIC less than 6 as a criteria [48,49]. Both STRUCTURE and DAPC analyses were conducted on the whole dataset (considering localities, habitats and trapping sessions) on the one hand, and on subpopulations sampled either inside houses or outside villages on the other hand.

### Ethics statements

This study was conducted as part of the national program activities, specifically within the murine surveillance system aimed at controlling plague reservoirs in an endemic focus, therefore, no permit was required. Verbal authorization from the community’s authority and village heads, as well as verbal consent from the owners or tenants of investigated houses and fields were obtained after informing them about the project. Outside villages, traps were always set on the edge of cultivated fields, so as not to damage crops. Small mammals were treated in a humane manner and manipulated by authorized and well-trained staff from the Plague Unit (Institut Pasteur de Madagascar) in accordance with guidelines of the American Society of Mammalogists [50] and the directive 2010/63/EU of the European Parliament. In addition, all procedures used here for capture, autopsy and sampling of wild small mammals were validated by Institut Pasteur de Madagascar Animal Ethics Committee (agreement 001/2025/IPM/DS/CEA).

## Results

A total of 2,762 *R. rattus* were captured during the two years of sampling in the six localities of the Ankazobe district (see [12]). Among them, 480 individuals were genotyped for the purpose of the present study, corresponding to 11–40 individuals per habitat (inside houses vs. outside villages), locality and session, thus forming 22 subpopulations (Table 1). Rats captured inside houses during March – May 2020 in Antakavana and Ambolotarakely were excluded from the dataset due to low sample sizes (n = 6 and n = 7, respectively) (Table 1) (Fig 1).

We excluded the locus D5Rat83 of the dataset because 49 individuals (10%) showed null genotypes following amplification problems. All other loci were found at HW equilibrium after correction for multiple testing, except D11Rat56 that displayed significant heterozygote deficiencies in several subpopulations, probably due to null alleles. The estimated mean frequency of null alleles at this locus was 20%, which may slightly impact genetic diversity and differentiation estimators [51]. We verified for most analyses that results were similar with or without this locus, and presented in the following the results obtained using this locus. After correction for multiple testing, LD was not significant in any of the tests performed. So, the 17 loci were considered to be independent.

Allelic richness (*r*) ranged from 5 to 6.3 alleles (mean 5.9 ± 0.1), and expected heterozygosity (*H*_*E*_) from 0.65 to 0.74 (mean 0.71 ± 0.01) (Table 1). Mean *F*_*IS*_ values ranged from 0.03 to 0.08 (Table 1). Genetic diversity estimates were significantly different among habitats, with subpopulations outside villages being slightly more diverse than subpopulations inside houses (*r*: *p* = 0.001; *H*_*E*_: *p* = 0.01; no significant difference for *F*_*IS*_*: p* = 0.27). Relatedness levels were higher (*p* = 0.004) within subpopulations inside houses (0.084) than outside villages (0.042). Genetic diversity and relatedness estimates were not different among sampling sessions (*r*: *p* = 0.75; *H*_*E*_: *p* = 0.29; *F*_*IS*_*: p* = 0.57; relatedness: *p* = 0.9).

**Fig 2:**
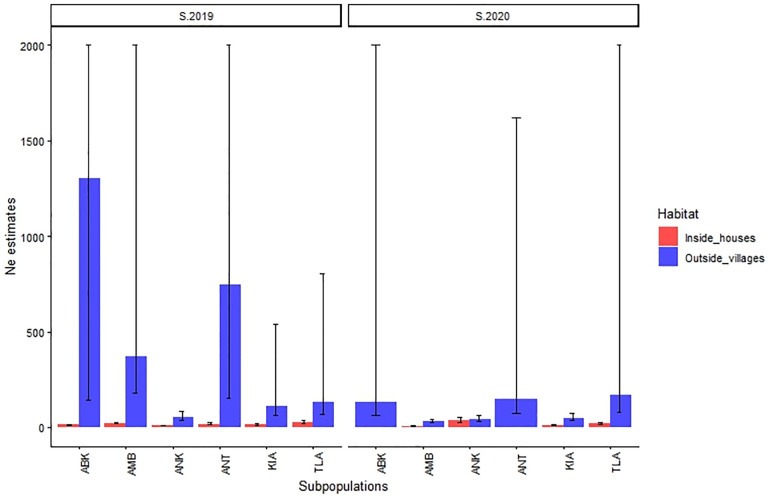
Mean *N*_*e*_ and its standard deviation, calculated using the LD method, in each subpopulation of *Rattus rattus* sampled inside houses and outside villages of the six localities during two trapping sessions (S.2019 and S.2020). ABK: Ambolotarakely; AMB: Ambohitromby; ANK: Ankazobe I; ANT: Antakavana; KIA: Kiangara; TLA: Talata-Angavo.

Effective population size *N*_*e*_ values were lower for subpopulations sampled inside houses (ranging from 6 to 35) than for subpopulations sampled outside villages (ranging from 33 to 1,301), with an overall mean of 157 ± 178 (Kruskal Wallis test: χ^2^ = 15.135, df = 1, *p* < 0.001) (Fig 2).

Seropositive rodents occurred in very low numbers in sessions and habitats analysed (Table 1). The overall plague seroprevalence across all localities and sessions was accordingly low (0.4%), ranging from 0.2% to 0.71% (Table 1). No significant correlation was observed at the locality level between plague seroprevalence (calculated from our dataset of 2,762 small mammals trapped over the entire 2-year long survey) and mean *H*_*E*_ (Spearman’s ρ = −0.25*, p* = 0.65), *r* (ρ = 0.08, *p* = 0.91) and *F*_*IS*_ (ρ = 0.49*, p* = 0.32).

The mean *F*_*ST*_ value computed across all subpopulations was 0.032 (95% CI = [0.028; 0.037]). Pairwise *F*_*ST*_ estimates ranged from −0.0016 to 0.0818 among all subpopulations. They were significantly higher among subpopulations sampled inside houses (*F*_*ST*_ = 0.045, 95% CI = [0.040; 0.053]) than among subpopulations outside villages (*F*_*ST*_ = 0.023, 95% CI = [0.017; 0.028]) (*p* = 0.007), but similar among subpopulations sampled within individual sessions (see S2 Table for all pairwise *F*_*ST*_ values from both inside houses and outside villages subpopulations). The AMOVA analysis showed significant genetic differentiation between localities (*Va* = 2.08%; *p* < 0.001) although most of the genetic variation was observed within individuals (*Vc* = 90.46%; *p* < 0.001). There was also significant genetic differentiation among habitats within localities (unweighted mean *F*_*ST*_ = 0.095; generalized binomial test *p* < 0.001). Genetic differentiation among sessions within localities was lower but also significant (unweighted mean *F*_*ST*_ = 0.003; generalized binomial test *p* < 0.001).

Genetic IBD was not significant among subpopulations at the scale of the district (slope *b* = −0.001, 95% CI = [−0.005, 0.003]) and when considering only subpopulations sampled outside villages (slope *b* = 0.007, 95% CI = [−0.010, 0.029]). However, there was a tendency towards a positive IBD when considering only subpopulations sampled inside houses (slope *b* = 0.058, 95% CI = [0.02, 0.098]).

**Fig 3:**
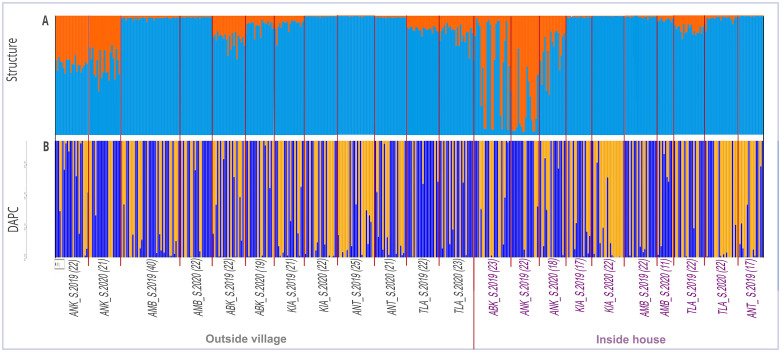
Genetic structure of *Rattus rattus* for all subpopulations (outside villages and inside houses, 2019 and 2020 sessions) of the Ankazobe district analyzed with STRUCTURE (A) and DAPC (B) with K = 2. In the barplots, each individual is represented by a vertical bar with the colors showing the proportion of the individual genotype derived from respective genetic clusters. ANK: Ankazobe I; AMB: Ambohitromby; ABK: Ambolotarakely; KIA: Kiangara; ANT: Antakavana; TLA: Talata-Angavo.

**Fig 4:**
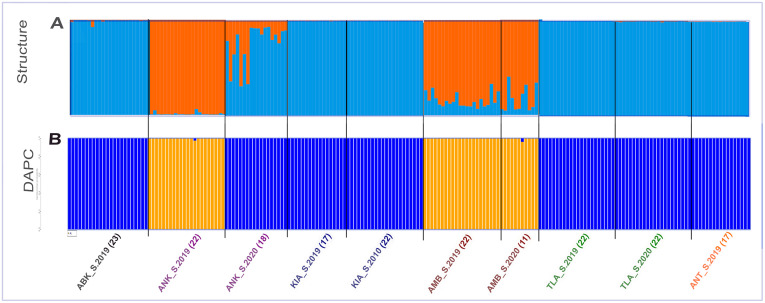
Genetic structure of *Rattus rattus* for all subpopulations sampled inside houses of the Ankazobe district, analyzed using STRUCTURE (A) and DAPC (B). See legend of Fig 3 for more information.

STRUCTURE was used to investigate whether rat populations were spatially structured in Ankazobe district. The highest *ΔK* value (26.87) was rather low, indicating no substantial substructure at the scale of the district (Fig 3A). When only subpopulations inside houses were considered, a weak differentiation was observed between the localities Ambohitromby (AMB) and Ankazobe I (ANK) subpopulations compared to the four localities (Kiangara, Antakavana, Ambolotarakely and Talata-Angavo) (Fig 4A). Alternatively, no substructure was observed when only subpopulations outside villages were analyzed. The DAPC analyses confirmed the results obtained with STRUCTURE (Figs 3B and 4B).

## Discussion

In Madagascar, in addition to the urban foci in the surroundings of Mahajanga seaport (West coast) [52,53], two main long-lasting plague foci have been described: the first one in the northern central area of the country, and the second one in the Central Highlands of Madagascar [11]. Ankazobe district is part of the latter one where plague remains endemic with suspected human plague cases being detected every year [54,55]. Previous studies have shown that *R. rattus* is the primary reservoir of *Y. pestis* and triggers human outbreaks in this district [12,55]. They were consistent with those of previous studies demonstrating the implication of *R. rattus* as the reservoir of plague in the Central Highlands of Madagascar [11,13].

The two-year surveillance study, including the two trapping sessions analyzed here, revealed low seroprevalence of *Y. pestis* in small mammals across the six localities [12]. However, human plague cases were regularly recorded during the same period (2019–2020) in other localities of the district. As such, from 2019 to March 2020, a total of 19 cases were reported, of which seven were confirmed at the Central Laboratory for Plague of the Ministry of Health (Unpublished data, Central Laboratory for Plague, Institut Pasteur de Madagascar). Low seroprevalence levels could be related to the fact that a substantial proportion of *R. rattus* from the Central Highlands was shown to be resistant to plague, some of them (up to 30%) remaining seronegative after experimental exposure [56,57]. Previous studies conducted in other plague-endemic regions of Madagascar also reported a low number of seropositive rats, even despite frequent human outbreaks, which was interpreted as consequence of their plague resistance [20].

Genetic diversity estimates were relatively homogeneous among subpopulations of rats in the six localities and between both sessions. They were relatively high (mean *r* = 5.9, mean *H*_*E*_ = 0.71) when compared to those observed in other African settings, for instance in Cotonou, Benin (*r* = 4.4, *H*_*E*_ = 0.58: [58]), in Niamey, Niger (*r* = 3.4, *H*_*E*_ = 0.5: [27]) or in Dakar, Senegal (*r* = 3.9, *H*_*E*_ = 0.67: [59]). Nevertheless, they were similar to those observed in other localities of the Central Highlands of Madagascar (mean *r* between 4.8 and 5.5, and mean *H*_*E*_ between 0.68 and 0.72 in four other highland areas [20]. Such differences between Malagasy and other African populations of black rats may suggest contrasted population sizes, which would be larger in Madagascar than in the other continental areas where the black rat is restricted to the commensal environment (Cotonou, Dakar and Niamey) and sometimes distributed in spatial patches (e.g., Niamey). They may also reflect differences in bio-invasion history, the black rat having been introduced much earlier in Madagascar (probably from the 11^th^ century: [60]) than in West Africa (probably during the European colonization era: [59,61]).

Genetic diversity was slightly higher for subpopulations outside villages compared to those from inside houses. This difference was consistent with that observed for effective population sizes (*N*_*e*_). The larger effective population sizes of subpopulations outside villages may reflect the much larger and continuous habitat, while habitat inside villages is of limited size (i.e., houses and their immediate surroundings). The temporal stability of genetic diversity estimates over one year (which may represent up to 2–3 generations of rats) and their lack of correlation with plague seroprevalence (as evaluated at the locality scale) suggests that plague epizootics, if any, did not leave any significant effect on population sizes. Alternatively, this impact may be too limited in intensity (relative to population size) or in time length, to impact genetic diversity [62].

Genetic differentiation among *R. rattus* subpopulations was globally low, indicating dispersal of individuals at the scale of the Ankazobe district, and/or large population sizes. This is consistent with previous results obtained in four other areas of the Malagasy highlands, reporting globally low genetic differentiation levels in black rat populations at similar spatial scales, which however increased with topographic relief [20]. Despite this low level of differentiation, our results suggest that subpopulations inside houses and outside village are differentiated within one given locality (unweighted mean *F*_*ST*_ = 0.095), indicating limited effective dispersal among habitats, as already suggested by previous studies in other Malagasy plague foci [19,20]. Limited movements of black rat individuals between both habitats would be consistent with the observation of different fleas on rats sampled in fields (mainly parasitized by *S. fonquerniei*) compared to those sampled within houses (infested by *X. cheopis*) [63]. Seasonal reproduction outside villages could imply longer-distance movements of individuals living inside this habitat compared to those living inside houses, thus potentially explaining lower genetic differentiation among their subpopulations. In contrast, individuals living inside houses would have less need to move in order to find mates or food, thus leading to slightly lower mobility. Higher relatedness levels of the subpopulations sampled within houses compared with those sampled outside villages are consistent with this interpretation. This hypothesis is also supported by the study of Rahelinirina et al*.* (2010), which used Rhodamine B to mark rats and track their movements in rural Madagascar: they showed that marked rats inside houses were primarily recaptured near their initial marking points [64].

The weak genetic differentiation found by STRUCTURE and DAPC analyses in subpopulations sampled inside houses may reflect isolation by distance, which may be only marginally significant due to the limited number of sampled localities. Alternatively, it could be driven by differences in population spatio-temporal dynamics. The localities ANK and AMB are more directly connected by frequent human movements to other major urban centers such as Antananarivo and Mahajanga than other localities of the study area, and could thus be more likely colonised by rats coming from these localities outside the Ankazobe district. Also, ANK and AMB localities reported higher numbers of human plague cases compared to others localities of the district between 2014 and 2018 (Unpublished data, Central Laboratory for Plague at IPM). Such high numbers of human cases may have been preceded by plague epizootics and subsequent recolonization of local populations by black rat individuals from different origins.

Finally, population genetics data can provide a biologically informed framework for defining management/eradication units and designing control strategies [65]. In Madagascar, rodent control is usually implemented locally from March to August, before the plague season, with the aim of reducing the number of breeding individuals and preventing subsequent population growth. Although black rats have a high reproductive rate and short generation time, with marked seasonal fluctuations of populations living outside villages, our results reveal a marked temporal stability of genetic structure across two consecutive years and strong genetic connectivity among villages at the scale of the district. Rat populations thus appear to function as a persistent and demographically connected system rather than as small, independently fluctuating units, leading to population stability at the district scale. The observed spatial connectivity implies that localized and temporally restricted control campaigns, if implemented at the scale of single villages, could be rapidly counterbalanced by dispersal from neighbouring areas. In this context, an integrated approach combining rodent as well as flea control within houses, together with environment-based management towards reduction of human-rodent contact (e.g., by securing food storage in elevated silos or separate sheds away from houses) may appear as a much more appropriate strategy to limit plague circulation among rats, and plague transmission from rat to human.

## Conclusion

This study revealed that *R. rattus* populations in Ankazobe district exhibit high genetic diversity, low differentiation and inter-annual stability. Significant differentiation between subpopulations sampled inside houses *vs.* outside villages reflect different spatio-temporal patterns in the two types of habitats. Altogether, our results questioned the efficiency of local rodent control when used alone to fight against plague. Rather, they advocate for more integrated strategies taking into account rodents, flea vectors and rodent-flea-human contacts.

## Supporting information

S1 Table Details of microsatellites markers used in this study. This table lists the microsatellite markers used in this study, including forward and reverse primer sequences with their associated fluorescent dyes, expected fragment sizes (in base pairs: bp), and pooling groups (Marker ID pooling) used for multiplex PCR amplification.
(XLSX)

S2 Table Pairwise *F*_*ST*_ values between subpopulations sampled both inside houses and outside villages subpopulations and their distributions represented in boxplot graph. This table provides all pairwise *F*_*ST*_ values between subpopulations sampled inside houses and outside villages as two diagonal matrices. Significant values according to the pairwise differentiation test performed in FSTAT are indicated in bold.
(XLSX)
