# Supplementary material for: Genetic structure of Rattus rattus populations in an endemic plague focus in Madagascar: Implications for rodent surveillance and management
Source: PLoS One. 2026 Jul 13;21(7):e0334819. doi: 10.1371/journal.pone.0334819 (PMC13362129; doi:10.1371/journal.pone.0334819)
Supplement: Attachment — Submitted filename: Response to Reviewers.docx [file pone.0334819.s004.docx]

**We thank the reviewers for their constructive comments. We carefully addressed all points raised. Detailed responses to each comment are provided below:**

**Reviewer comment:** ‘microsatellite’ in the keywords should be in plural with regards to the plurality of biomarkers, or ‘microsatellite markers.

**Author:** we have corrected the keyword to “microsatellite markers” (l. 40).

**Reviewer comment:** **Abstract**: As you have focused the paper on data from two trapping sessions, one in March-May 2019 and another in March-May 2020, is ‘temporal structure’ still relevant? Does significant change of genetic structure occur in such duration?

**Author:** we agree that the terms “temporal structure” may be misleading. We modified the sentence as: “this study aims at investigating the genetic structure of *R. rattus* populations at a fine geographical scale and across two different years” (l. 22-23).

**Reviewer comment:** Change ‘Our results show’ to ‘…. showed’.

**Author:** The correction was made in the revised version (l. 27).

**Reviewer comment:** ‘… combining flea control within houses’ may be ‘… flea control inside houses’?

**Author:**  the correction was made in the revised version (l. 36).

**Reviewer comment:** **Introduction**: ‘… in close contact with people’ => ‘… with humans’?

**Author:** the correction was made in the revised version (l. 49).

**Reviewer comment:** ‘central highlands’ to ‘Central Highlands’.

**Author:** the correction was made in the revised version (l. 55-56).

**Reviewer comment:** ‘above 800 m high’ to ‘at elevation above 800m’.

**Author:** the correction was made in the revised version (l. 56).

**Reviewer comment:** ‘to the relief in the central highlands’ => ‘…. Central Highlands’.

**Author:** the correction was made in the revised version (l. 70-71).

**Reviewer comment:** ‘inside village houses’ => ‘inside houses’.

**Author:** the correction was made in the revised version (l. 79).

**Reviewer comment:** **Materials and methods**: ‘central Highlands (mean altitude of 1,190 m)’ => ‘Central Highlands…’

**Author:** the correction was made in the revised version (l. 85).

**Reviewer comment:** ‘altitude’ => ‘elevation’.

**Author:** the correction was made in the revised version (l. 85).

**Reviewer comment:** ‘namely: Ambohitromby’ => ‘namely Ambohitromby’.

**Author:** the correction was made in the revised version (l. 86).

**Reviewer comment:** ‘Hills slopes’ => ‘Hill slopes’.

**Author:** the correction was made in the revised version (l. 90).

**Reviewer comment:** Trapping sessions: as you are focused on two trapping sessions, it may not be necessary to mention ‘Six small TS…’

**Author:** this part of the text was reworded (l. 99-104), in order to focus on the two trapping sessions that were considered.

**Reviewer comment:** Specify meaning of ‘night-traps’ (calculation) to ease understanding of 3,888 going from 18 houses during three nights.

**Author:** we agree that the total number of 3,888 night-traps was confusing, especially since it referred to the six initial trapping sessions among which only two were considered in this manuscript. We removed indication of the total number of night-traps and clarified the trapping design by specifying the number of traps, houses, and nights only (l. 108-110).

**Reviewer comment:** Is there any particular reason why trapping efforts were different between habitats? 18 houses (36 traps?) against 84 traps outside villages.

**Author:** we thank the reviewer for this comment. The difference in trapping effort between indoor habitats and outside villages was in part due to logistical and ethical constraints: indoor trapping could only be conducted in houses where the owner or tenant had provided verbal consent. Importantly, our objective was not to compare trap success between indoor and outside village habitats, but rather to collect sufficient numbers of individuals from each habitat in each locality to perform the population genetic analyses. For this reason, we believe that the difference in trapping effort has a limited impact on the interpretation of our results.

**Reviewer comment:** ‘Species, sex, external measurements (head-body length, tail length, ear length and hind foot length, in mm) and weight (in g) were recorded on each individual.’ You can arrange sentence to say that body mensuration (or external measurement) allowed identifying rodent at species level.

**Author:** The sentence was modified as suggested by the reviewer (l. 115-120).

**Reviewer comment:** Kidney of each individual was sampled then stored in 95° ethanol for subsequent molecular investigation purpose. Furthermore, blood was collected by cardiac puncture and centrifuged to obtain sera samples for serological analyses. I think blood sampling goes before kidney sampling?

**Author:** the sentences have been modified to indicate that blood was collected before to kidney sampling (l. 120-124).

**Reviewer comment:** For each individual, total DNA was extracted from kidney using QIAamp DNA Mini Kit (Qiagen, Hilden, Germany) as recommended by the supplier. Manufacturer instead of supplier.

**Author:** the correction was made in the revised version (l. 132-133).

**Reviewer comment**: A table showing microsatellite marker features would be helpful. How 18 markers were pooled during multiplex reactions? What is the expected amplicon size per locus? Here there are 18 markers whereas in the abstract, there are 17. Also, can authors explain the use of markers developed for brown rat to test black rat? Isn’t it required to use species-specific microsat markers?

**Author:** a table showing microsatellite marker features, expected amplicon size per locus, and marker ID pooling information was provided as Supplementary Material in the revised version (S1 Table) (l. 137).

Individuals were genotyped using 18 markers, but one of them was excluded from the analysis given the number of null genotypes. We corrected the abstract for more clarity.

Regarding the use of markers developed for brown rat (*Rattus norvegicus*), these loci have been successfully applied to black rat (*R. rattus*) in previous studies, showing reliable amplification and sufficient polymorphism (Brouat *et al*., 2013; Brouat *et al*., 2014; Berthier *et al*., 2016; Badou *et al*., 2023).

**Reviewer comment:** ‘Detection was conducted using an ABI 3130xl Genetic Analyzers (Applied Biosystems, Foster City, CA, USA) and genotypes were red, analysed…’. Detection of ….? Read instead of red?

**Author:** the sentence was modified as following: “Genotyping was performed with an ABI 3130xl Genetic Analyzers (Applied Biosystems, Foster City, CA, USA) and microsatellite profiles were read, analysed and eye-cleaned under GeneMapper v.6 software.”(l. 139-141).

**Reviewer comment:** ‘Each group of samples from a given habitat × locality × session was considered as a distinct subpopulation in the subsequent analyses.’ You can change to ‘Individuals belonging to a given habitat (inside house or outside village), locality (the same site?), and sampling session (2019 or 2020) were considered as a distinct subpopulation in the subsequent analyses’

**Author:** the sentence was modified as suggested in the revised version (l. 144-146).

**Reviewer comment:** ‘Nei’s unbiased genetic diversity (HE, [28]) and FIS’. Change to ‘………….. and the inbreeding coefficient FIS’.

**Author:** the correction was made in the revised version (l. 154).

**Reviewer comment:** Can authors spell out LD methods?

**Author:** the correction was made in the revised version (l. 155-156).

**Reviewer comment:** For IBD analysis, please specify what is considered as subpopulation? Still pop from a habitat?

**Author:** yes, subpopulation is used as described above in the manuscript, and as clarified by the reviewer (see above).

**Reviewer comment:** ‘Rats captured inside houses during March-May 2020 in Antakavana and Ambolotarakely were excluded from the dataset due to low sample sizes (n = 6 and n = 7, respectively) (Fig 1).’ You can also cite Table 1 in addition to Fig1.

**Author:** citation of Table 1 was added in the revised version (l. 217).

**Reviewer comment:** Table 1: HS meaning?

**Author:** “*HS* in Table 1 was corrected to *H_E_* (expected heterozygosity).

**Reviewer comment:** **Results**: ‘STRUCTURE was used to investigate whether rat populations where spatially structured in Ankazobe district.’ Were instead of where.

**Author:** the correction was made in the revised version (l. 270).

**Reviewer comment:** **Discussion**: ‘During our 2 year-long survey’. If I’m not wrong, samplings were performed once per year during two years.

**Author:** we clarified the sentence to indicate that the two-year surveillance study, including the two trapping sessions analyzed here, revealed low seroprevalence of *Y. pestis* in small mammals across the six localities (Parany *et al*., 2025) (l. 299-302).

**Reviewer comment:** As the genetic diversity varied according to habitat (higher outside village), how is the connection/gene exchanges between populations from inside houses with those from outside? In the bibliography, rat would move from field to inside houses following harvest.

**Author:** our results indicated low but significant differentiation between rat populations inside houses and outside villages. This differentiation suggests limited effective dispersal among habitats, but movements of individuals may occur regularly. The observed difference in genetic diversity could be explained by differences in population size.

**Reviewer comment:** ‘the observation of different ectoparasites on rats’ change to ‘… different fleas…’

**Author:** the correction was made in the revised version (l. 344).
