# Supplementary material for: Genetic structure of Rattus rattus populations in an endemic plague focus in Madagascar: Implications for rodent surveillance and management
Source: PLoS One. 2026 Jul 13;21(7):e0334819. doi: 10.1371/journal.pone.0334819 (PMC13362129; doi:10.1371/journal.pone.0334819)
Supplement: Attachment — Submitted filename: Response to Reviewers_2.docx [file pone.0334819.s005.docx]

**We thank the reviewers for their constructive comments. We carefully addressed all points raised. Detailed responses to each comment are provided below:**

**Reviewer #1**: My previous comments are addressed by authors. The revised version was highly improved.
I just have one comment/question:
Lines 125-126: eight or seven markers?

**Author:** The number of microsatellite markers developed for *R. norvegicus* is eight: the D5Rat83 was added in the marker list in the revised version (**l. 159** in the revised version with tracked changes).

**Reviewer #2**: This is my first review of this manuscript. I understand it has undergone previous review, and I acknowledge the work done to reach this stage. The manuscript is clear and the results are relevant for pest control.

However, my main question relates to the clarity and justification of the research objective concerning the temporal genetic structure between the two sampling years, which are only one year apart. The introduction does not sufficiently establish why investigating genetic changes from one year to another (2019 vs 2020) is a worthwhile question for this system.

**Author:** we thank the reviewer for this comment and agree that the objective required clarification. We have revised the Introduction accordingly (**l. 83-97** and **101-108**). Our objective in comparing 2019 and 2020 samples was to assess the short-term temporal stability of fine-scale genetic structure, rather than to investigate long-term evolutionary change. Black rats have short generation times (about 6 months) and high reproductive rates, so a one-year interval may encompass different generations. In addition, rat populations in the Central Highlands undergo marked seasonal fluctuations in abundance and may be affected by plague-induced mortality and human-mediated dispersal among villages. These factors could theoretically lead to rapid shifts in allele frequencies at a local scale. Testing temporal stability over one year is therefore important to determine whether the observed spatial genetic structure reflects persistent local populations or transient demographic dynamics. Demonstrating stability (or instability) directly informs the interpretation of gene flow and connectivity among villages, and is thus important regarding the implementation of plague control measures.

**Reviewer #2**: See specifics below:

Ln68: Could you please elaborate on these previous results, particularly their spatial extent? While the importance of the spatial scale is clear, the relevance of the temporal scale is less obvious. What is the generation time of the rat population? Is the consideration of time primarily due to seasonal variations in rat abundance? Or, is it because rats are routinely exterminated within houses each year, leading us to expect that rat colonization events are an annual occurrence? Please make clear justification of the objectives regarding the temporal question. Relying on the reader to piece it together from the introduction and methods reduces the text's clarity and impact.

**Author:** we agree with the reviewer, and have elaborate on previous results in this part (**l. 83-97**). As explained just above, we have also clarified why we aim to describe the temporal genetic structure (**l. 101-108**).

**Reviewer #2:** Ln97-99: In terms of distance, what average distance was from inside the house’s vs outside of the village? The distance between villages is provided, but which were the min. and max. distances between the two sampling habitats? This is relevant to interpreting the results.

**Author:** within localities, the minimum distance between sampling habitats (inside houses vs. outside the village) was 11.4 m, the maximum distance was 234 m, and the mean distance was 106 m. These distances were calculated using GPS coordinates with the R package geosphere, representing straight-line distances. The real paths used by black rats may differ due to landscape features.

This information has been added in the revised version (**l. 128-129**).

**Reviewer #2:** Table 1. Session S1 and S2 relate to the two sampling years? Why not instead to put the years S2019, S2020

**Author:** the labels “S1” and “S2” were replaced with “S.2019” and “S.2020” in the Table 1 and throughout the revised manuscript and in figures.

**Reviewer #2:** L223: among sampling sessions

**Author:** the correction was made in the revised version (**l. 263**).

**Reviewer #2:** Ln265-268: I don’t have clear why genetic structure was analyzed separately for inside and outside villages?

**Author:** we thank the reviewer for this comment. We analyzed genetic structure separately for individuals sampled inside houses and outside villages because these two habitats represent distinct ecological contexts that may influence rat population dynamics and connectivity. Indeed, rats living inside houses benefit from more stable food resources and shelter, and may reproduce continuously. As a result, indoor populations may function as relatively discrete demographic units, potentially more resistant to immigration from surrounding outdoor populations. In contrast, rats living outside villages reproduce seasonally, and may experience increased dispersal among populations during periods of low density, likely associated with greater spatial connectivity, thereby forming a more continuous population across the landscape. We therefore hypothesized that genetic structuring might be more detectable among house-dwelling subpopulations than among outdoor individuals. Separate analyses were conducted to test this ecological hypothesis and to assess whether habitat type influences fine-scale genetic patterns relevant for plague control strategies. We have clearly stated this hypothesis in the Material and method (**l.** **221-225**).

**Reviewer #2:** Ln317: this information should be in the introduction, I have still no clarity of why comparing two sampling years.

**Author:** information about generation time was added in the introduction section of the revised version (**l. 93-94**), and the objective to compare two sampling years has been made more explicit (see above).

**Reviewer #2:** Ln359-362: Expand further the management recommendation provided; I don’t see much difference between what is being done and what is being recommended. Again, the initial question regarding year-to-year differences is not clear, and not linked to a specific biology of the species or related to a particular pest control management.

**Author:** as clarified in the introduction of the revised version (**l. 83-97 and l. 101-108)** and in the last paragraph of the discussion related to management recommendation (**l. 400-414**), the temporal analysis was designed to clarify the biological meaning of the observed spatial genetic structure. Specifically, comparing two consecutive years allowed us to determine whether the spatial differentiation detected among villages reflected stable population structuring or transient patterns driven by rapid turnover and recolonization dynamics. The absence of significant temporal genetic differentiation indicates that allele frequencies and clustering patterns are stable over time, supporting the interpretation that rat populations at the district scale form a persistent and genetically connected system rather than ephemeral, independently fluctuating units. This temporal stability therefore strengthens the inference drawn from spatial analyses, namely that villages do not constitute isolated management units. We hope that it is clearer now in the revised version.

**Reviewer #3:** Body of work presents technically sound original research.
Would be nice to see comparisons made to somewhat similar work published in the Canadian Journal of Zoology, 2007. This work feels like a scale up of what was done there?

**Author:** we thank the reviewer for this suggestion. The comparison with Gilabert et al., 2007 is now explicitly made in the revised version (**l. 373-377**).

**Reviewer #3**: Transparency is important and I feel the authors should share how the analyses were done, instills confidence that the work was done as written and imparts knowledge to others.

**Author:** we thank the reviewer for emphasizing the importance of transparency. All analytical procedures are described in detail in the methods section to ensure reproducibility and clarity. In addition, the genotype dataset has been made publicly available in the Zenodo repository at the following link: <https://doi.org/10.5281/zenodo.17810727> and details of microsatellites markers and primer sequences with their fluorescent dyes, expected fragments and pooling group of markers for multiplex PCR are available in the supporting information.

**Reviewer #3:** Microsatellite genotyping section - please indicate temporal distinction of the samples genotyped. Also, based on the rates of evolution for some molecular marker's, I am not sure one can expect to see temporal difference with microsats over a year's time, even if you managed to catch the same individual?

**Author:** sample size per locality, habitat and session are specified in Table 1: we have clarified the reference to this table in the revised version, and added references to sampling sessions (**l. 265-266**). We agree that the rationale for comparing two temporally close sampling years was not sufficiently explained in the original version, a concern that was also raised by Reviewer 2. Regarding the evolution rate of microsatellites, previous studies already reported temporal variation in the genetic structure of black rat populations over short time intervals using microsatellites markers (for instance, see Berthier *et al.* 2006 in Mol. Ecol.; Kajdacsi *et al.* 2023 in Mol. Ecol.). We hope that the revised version and the detailed responses above satisfactorily address the reviewer’s comments.

**Reviewer #3:** Data analyses section - please indicate what tools what were used at the very start or first few lines of the paragraph. Also, may just mention 'R v 4.4.2' instead of 'R software version....'.

**Author:** as we used several software and tools, we propose to keep the mention of each software immediately after the corresponding test or analysis to avoid confusion. The R software version is specified in the revised version (**l. 172**).

**Reviewer #3:** Not sure non-parametric tests will give anything remotely definitive on plague leaving a trace on genetic diversity? unless the objective is to just give an association? somewhat superficial relationship? Certain you are aware non-parametric tests don't provide a cause-effect relationship.

**Author:** we thank the reviewer for this comment. Our objective in using these tests was solely to explore potential associations between the presence of plague and patterns of genetic diversity, rather than to infer a cause-effect relationship. The sentence was modified to clarify this point in the revised version (**l. 182-187**).

**Reviewer #3:** Line 183: did not see mention of STRUCTURE and DAPC analyses being used to test temporal variation? unless I missed whether this was stated?

**Author:** global STRUCTURE and DAPC analyses, including all sites, habitats, and trapping sessions, were indeed performed. These analyses revealed no signal for temporal genetic structure, and given these results, we did not conduct separate analyses by trapping session (S.2019 and S.2020). In addition, unlike for the spatial analyses, we did not formulate hypotheses that could explain temporal variation.

**Reviewer #3:** In my experience STRUCTURE and DAPC rarely give you similar answers, always a '1 K' margin of error...of course this is dependent on inherent differences in the marker of choice and algorithmic biases...please review all mentions of DAPC as confirmatory analyses and suggest that changes to them being secondary analyses.

**Author:** we have revised the manuscript to present DAPC analyses as a confirmatory analysis, individual-based approach to explore population structure (**l. 215-216**).
