# Supplementary material for: Genetic structure of Rattus rattus populations in an endemic plague focus in Madagascar: Implications for rodent surveillance and management
Source: PLoS One. 2026 Jul 13;21(7):e0334819. doi: 10.1371/journal.pone.0334819 (PMC13362129; doi:10.1371/journal.pone.0334819)
Supplement: Attachment — Submitted filename: Response to reviewer_3_Parany.docx [file pone.0334819.s006.docx]

**We thank the reviewers for their constructive comments. We carefully addressed all points raised. Detailed responses to each comment are provided below:**

**Reviewer #2:** I have read the revised version of this work and I found that the authors have addressed all my comments satisfactorily. I have no further comments.

**Author:** We thank the Reviewer #2 for revising this manuscript.

**Reviewer #4:** The manuscript is clear and well structured, and the objectives, methods, and results are presented in a coherent and understandable manner. The authors have adequately addressed the issues raised during the review process, and the revised version meets the standards for publication. The study contributes relevant data to the understanding of rodent population dynamics in relation to disease surveillance.

I have no additional comments or concerns regarding research ethics or publication ethics. I support the publication of this manuscript.

**Author:** We thank the Reviewer #4 for his/her time and support.

**Reviewer #5:** This is my first revision of Parany et al. The study aims at quantifying genetic structure of rat populations occurring inside houses and outside houses across the Ankazobe district in the Central Highlands of Madagascar. This study is relevant in the context of plague outbreak, since rats are one of the major reservoir of plague spread. The authors used 17 microsatellites loci of >400 individuals sampled over two seasons (March-May 2019 and March-May 2020) to investigate genetic diversity, effective population size and genetic structure inside and outside houses, and across sampling seasons. They found higher genetic diversity and effective population size in the outside houses populations compared to the inside houses. There are also evidence of stronger genetic structure in the inside house populations. Overall, they conclude that the higher genetic diversity in the outside populations is likely due to the fact that there is higher dispersal among outside populations than inside populations. Higher dispersal in the outside populations suggest that rodent surveillance plans should not carry out on single subpopulations, but considering the whole network of rat connectivity.

The manuscript is well written and clear. I think that they also present well the context. That being said I am not an expert on disease spread, plague and rat-plague co-dispersal so I cannot judge if the state of the art of the topic has been accurately presented.

I think that the authors have a quite unique dataset, although currently limited to microsatellites. I have some questions and suggestions that may help increase the impact of this work. I suggest the publication of this work upon minor revision.

**General Comments**

I might have missed it but it would have been interesting to see a quantitative comparison of plague seroprevalence between the inside vs outside houses rat populations. If the authors have enough data to quantify such differences, it would also be interesting to assess whether differences in seroprevalence between inside and outside houses rats correlates with measures of genetic diversity (He and Fst) in the respective populations (inside and outside houses). Also, did the author computed seroprevalence per sampling seasons? Are there difference between those?

**Author:** Unfortunately, the number of seropositive individuals was very low in our dataset, thus greatly limiting statistical power and precluding robust comparisons between rats from inside versus outside houses: that is the reason why seroprevalence levels were just compared among localities. To make this point clearer, the number of seropositive rodents is now reported in Table 1 of the revised version.

Also, I noticed that the authors reported genetic IBD statistics for the inside houses populations and overall sampling, but not among outside houses rat populations. Did I miss it or there is a specific reason for not doing that?

**Author:** We agree with the reviewer. Although not significant, IBD statistics are now specified in the revised version for rat subpopulations outside houses (**Lines 302- 303**).

I think that in this context spatial analyses of genetic structure are quite relevant to understand how rat dispersal impact plague spread. I would suggest the authors to add few other spatial analyses which are related to genetic IBD but different, such as spatial autocorrelation or EEMS analysis (which measure rate of relative dispersal across a landscape using either microsatellite or SNP data) [https://github.com/dipetkov/eems].

**Author:** We thank the reviewer for this helpful suggestion and agree that spatially explicit analyses such as spatial autocorrelation or EEMS could provide valuable insights into rat dispersal and its potential role in plague spread. Both spatial autocorrelation analyses and EEMS generally require a relatively dense and spatially continuous sampling scheme to reliably infer patterns of gene flow across heterogeneous landscapes. In our study, sampling was limited to six sites located outside villages, which provides limited spatial resolution and does not adequately capture landscape continuity. In addition, our dataset includes 17 microsatellite loci, which, while sufficient for estimating population structure and genetic differentiation, may provide limited precision for spatially explicit inference of migration surfaces, particularly for methods such as EEMS that typically benefit from a larger number of genetic markers (e.g., high-density SNP datasets). For these reasons, we believe that applying these methods to our dataset would not yield robust or biologically interpretable results. Nevertheless, we acknowledge their relevance and agree that such approaches would be valuable in future studies with denser spatial sampling and higher-resolution genomic data.

**Detailed Comments (numbering refers to the newest version of the manuscript I received)**

Line 296: I think this sentence should be rephrased. The genetic IBD appears to be significant but negative in slope. I see what the authors means, but I think it should be rephrased.

**Author:** We agree that this sentence is misleading. The reported p-value (p = 0.001) results from a permutation-based test of the overall correlation structure, which can detect very weak statistical associations. However, the estimated slope of the relationship between genetic and geographic distances was extremely small and not significantly different from zero, as indicated by the 95% confidence interval overlapping zero. We therefore interpreted this result as indicating no biologically meaningful isolation by distance. To avoid confusion, we have deleted the reported p-values, in order to make clear that our interpretations were based on slopes and their confidence intervals (**lines 301 - 305).**

Line 297-299: what about genetic IBD for outside houses populations?

**Author:** The IBD for rat populations outside houses is not significant. We have now included this result in the revised manuscript (**lines 302 - 303).**

Line 282: I think it could be nice to better point to the south and northern populations in Fig. 3. I feel it is very difficult to see that at the moment.

**Author:** We thank the reviewer for this suggestion. However, we share the view of Reviewer #6 that it was probably overstated to interpret the genetic differentiation between ANK and AMB and the other four localities (KIA, ANT, TLA and ABK) as a north–south gradient. The discussion was thus modified on this point in the revised version (see below).

Line 358-360: replace “larger sizes” with “larger effective population size”. Higher genetic diversity or Ne do not directly translate into higher census size, but it might be related to patterns of dispersal, higher in the outside houses than inside houses populations (as also the authors points out in the discussion).

**Author:** this has been done (**line 365).**

Line 362-363: Could you measure relative abundance based on rat capture rate over the two sampling seasons and subpopulations?

**Author:** The results on relative abundances by habitat and by session are fully presented in Parany et al. (2026): these demographic data come from a longitudinal study (2018 – 2020) while the rats genetically analyzed in the present manuscript are only a part of them (2019-2020). Doing so, we were still able to relate the population genetics results to the trends observed in relative abundance across sessions. No significant difference in capture rates was observed between S2019 and S2020 sessions (March – May), indicating stable relative abundance over time. This pattern is consistent with the absence of temporal genetic structure observed in this study.

**Reviewer #6:** Reviewer's report: Genetic structure of *Rattus rattus* populations in an endemic plague focus in Madagascar: implications for rodent surveillance and management.

This is my first review of the Parany et al. manuscript and I can see the authors have been thorough in responding to comments of all previous reviewers. This exploratory study provides an interesting comparison of genetic structure among rat subpopulations sampled from two environments: inside houses versus surrounding fields. The authors make key inferences regarding the rapid recolonization of rats, which is significant for plague control efforts in Madagascar.

Overall, the population genetic analyses are robust at both district-wide and local scales. However, some of the inferences are overstated, such as interpreting the genetic differentiation of Ankazobe (ANK) and Ambohitromby (AMB) as being a north-south pattern. ABK (a southern location) does not support a north-south hypothesis, based on the data presented. An alternative explanation that seems more plausible is given in the Discussion; namely, proximity to major urban centers which could potentially lead to gene flow into ANK and AMB. I recommend removing text related to a north-south pattern in the Discussion, and instead focus on the finding that ANK and AMB are members of a genetic group that is distinct from the other four locations.

**Author:** We agree with the Reviewer for this point. ANK and AMB are localities that are more directly connected with frequent people interactions with other major urban centers such as Antananarivo and Mahajanga than the other four localities (KIA, ANT, TLA and ABK) of the district, and could thus be more likely colonised by rats coming from localities outside the Ankazobe district. We modified the corresponding sentences in Results (**Lines –309 - 319)** and Discussion sections (**Lines 394 - 403**).

Major Revisions:

1. **Introduction:** I saw the main hypothesis described in the Methods on lines 203-206. I think it would make the Introduction stronger if you also mentioned this hypothesis (briefly) in the last paragraph of the Introduction (perhaps after line 91). I especially liked the explanation you provided to Reviewer #2, along the lines of : “Because rats living indoors may function as relatively discreet demographic units (Rahelinirina et al. 2010), we tested the hypothesis that genetic structuring might be more detectable among house-dwelling subpopulations than among outdoor individuals.”

**Author:** We thank the Reviewer for this suggestion, we have taken it into account and have included this hypothesis in the Introduction in the revised version. **Lines 88 – 92.**

2. **Methods**: It would benefit your audience to briefly describe 2019-2020 rat control operations in Ankazobe district. Mention if rat control efforts had taken place at your specific study sites before trapping occurred.

**Author:** As recalled above, we conducted a longitudinal trapping survey across six localities in Ankazobe, with six trapping sessions. For the genetic analyses, we selected two of these sessions (S.2019 and S.2020). As specified in the revised version, no large-scale rat control operations had been conducted in our study sites prior - to trapping. (**Lines 116 – 117**).

3. **Methods:** Related to point#2, it would also be helpful to mention whether rat removal during your trapping efforts would be expected to significantly reduce rat numbers in residences and surrounding habitat. I am curious to know if rat removal could explain the lower Ne estimates inside houses.

**Author:** While our trapping removed some individuals, it was limited in scope and unlikely to have significantly reduced rat numbers in residences or surrounding habitats. A recent study conducted in Malagasy rural habitats (like those investigated here) using similar protocols showed that trapping inside houses did reduce rodent activity in the very short-term (a few weeks) with no effect on the longer-term, with rodent abundance in similar levels by the following month (Rahelinirina et al., 2021 - Integrative Zoology 2021; 16: 868–885). Therefore, the observed lower Ne estimates inside houses retrieved in our study likely reflect natural population structure rather than an artifact of rat removal.

4. **Methods:** Please add a few more details on the trapping design. Outside the village, you placed 84 traps spaced by 10 m. Were these placed in a single long transect 830m long, or multiple shorter transects? Or in a grid design? If a grid, please give the size of the grid area.

**Author:** We placed the 84 traps spaced by 10 m in a single long transect 830m long. We have added this detail in the **Lines 133-137**.

5. **Results Line 267:** One of the most important results is that genetic structure is higher among rat subpopulations sampled inside houses than among subpopulations sampled outside villages. Please add the two Fst values to this sentence (one for inside houses, one for outside villages) and the range of pairwise Fst values. In fact, it would help readers to see all pairwise Fst values from inside the houses in a table format (probably as a diagonal matrix). A separate diagonal matrix could be used for samples from outside villages. If the authors are willing to do so, these diagonal matrices could be added as supplementary material. This would give a clear understanding of variation among Fst values and show whether the pattern is driven by a few outliers, or is consistent across all pairwise Fst comparisons.

**Author:** Fst values for inside houses and outside villages subpopulations as well as the range of pairwise Fst values have been added in **lines 290 - 294**. Furthermore, we provided all pairwise Fst values from both inside the houses and outside villages as diagonal matrices in the S2 Supplementary Table (**lines 649 – 653**). Only one pairwise *F_ST_* value was not significant for pairs of subpopulations sampled inside houses, compared to ten values for pairs of subpopulations sampled outside villages (see Table S2). Moreover, a boxplot graph illustrates that the pattern of higher genetic structure difference among rat subpopulations sampled inside houses than among subpopulations sampled outside villages is not driven by a few outliers.

6. **Results:** Related to point#5 above, is the genetic structure of rats in houses due to relatives remaining in this habitat instead of dispersing? You could answer this by using FSTAT to run a “Comparison among groups of samples” procedure, and choose the Relatedness option to compare samples from inside houses vs. outside villages. This test would provide additional insight into the aspects of rat biology that shape genetic structure at a fine scale.

**Author:** We thank the Reviewer for this suggestion. We compared relatedness within subpopulations sampled inside houses and outside villages using FSTAT, and found higher levels within subpopulations sampled inside houses, as it is expected under the hypothesis of low dispersal. This analysis has now been included in the Methods, Results and Discussions sections (**Lines 181 – 182; 257 – 261; 388 - 390**)**.**

**7. Discussion** Line 385: What is meant by “environmental-based management towards reduction of human-rodent contact”? Please give 1 or 2 specific examples. (for instance, do you mean house improvements to prevent holes that allow rat access, or storing food in a shed separate from the living space?)

**Author:** We have added some examples in the Discussion section to clarify “environmental-based management towards reduction of human-rodent contact,” **Lines 421 - 422.**

**Minor Revisions:** The manuscript is generally well-written, but some revisions are needed for clarity. Here are my suggestions for re-wording specific sentences.

8. Line 22: Change to “In this context, our study investigates…”

**Author:** Done. **Line 22.**

9. Line 25: Change to “…and in habitat outside of villages”

**Author:** Done. **Lines 26-27.**

10. Line 28: Change to “…relatively similar among villages”

**Author:** Done. **Line 29**

11. Line 36: “Because of the re-colonization problem, an integrated approach…”

**Author:** Done. **Line 38**

12. Line 47: “within or near these natural foci”

**Author:** Done. **Line 49**

13. Line 78: “…which may be associated with the heterogeneous”

**Author:** Done. **Lines 80 - 81**

14. Line 90: …effective population size (Ne),

**Author:** Done. **Line 98**

15. Line 90: “and examined the short-term stability”

**Author:** Done. **Line 98**

16. Line 132: Remove “purpose”.

**Author:** Done. **Line 146**

17. Line 149: Change to “and manually corrected in GeneMapper…”

**Author:** Done. **Line 166**

18. Line 191: “genetic groups”

**Author:** Done. **Line 210**

19. Line 214: Change human to “humane”

**Author:** Done. **Line 228**

20. Line 237: I suggest replacing homogeneous with “similar” (but this is up to the authors)

**Author:** The sentence “Genetic diversity estimates were relatively homogeneous among subpopulations has been deleted to avoid confusion. **Lines 251 - 252**

21. Line 253-254: Remove the text in this sentence, it repeats the same information as the first sentence. Keep the statistical reporting by adding it to the first sentence.

**Author:** The sentence has been modified. **Lines 274 – 277**

22. Line 268: Replace “…in one session or in the other” with “…within individual sessions”.

**Author:** Done. **Line 292 – 293**

23. Line 272: after Fst = 0.095, add the range of pairwise values.

**Author:** Pairwise values of Fst are now reported in Table S2.

24. Line 288: use lower case for north-south.

**Author:** North-south expression has been deleted. **Line 318**

25. Line 326: change “in four areas” to “in four other highland areas”.

**Author:** Done. **Line 356**

26. Line 376-377: Remove the word “although” from the start of the sentence and place it after the first comma “, although with marked seasonal fluctuations, and our results reveal…”.

**Author:** The sentence has been modified. **Lines 412**

27. Line 379-381: I recommend merging these two sentences in the following way: At the end of the first sentence add “…fluctuating units, leading to population stability at the district scale. I would then remove the second sentence (Line 380-381) because the term “limited natural turnover” seems confusing in this context.

**Author:** Done. **Lines 416 - 417**

28. Line 386-387: Consider removing “on the one hand….on the other hand”.

**Author:** Done. **Lines 423 - 424**

29. Table 1: The order of subpopulations in the table is not the same as in Figure 2 (or Fig. 3). I suggest re-arranging the Table to match the order given in Figure 2 (ABK, AMB, ANK, ANT, KIA, TLA). This will make it much easier for your readers to follow the reporting of results.

**Author:** Done. **Lines –263 - 270**

30. Fig 1: Increase the size of the inset showing Madagascar, so that readers will be able to find Ankazobe.

**Author:** Necessary done.

31. Fig. 1 caption: Add a period after “district.” Also, end with “…two sessions (2019 and 2020).”

**Author:** Done.

32. Fig 2 caption: Change NE to “Ne”. Remove “s” from subpopulations

**Author:** Done.

33. Fig. 3 caption: add “s” to house. Spell out full name of *Rattus rattus* for all figure and table captions.

**Author:** Done.
