# Supplementary material for: Genetic structure of Rattus rattus populations in an endemic plague focus in Madagascar: Implications for rodent surveillance and management
Source: PLoS One. 2026 Jul 13;21(7):e0334819. doi: 10.1371/journal.pone.0334819 (PMC13362129; doi:10.1371/journal.pone.0334819)
Supplement: Peer review history

# Peer review: Decision Letter 0

**DOI:** 10.1371/journal.pone.0334819.r001

**Authors:** Tzen-Yuh Chiang

28 Nov 2025

PONE-D-25-53545

Genetic structure of *Rattus rattus*  populations in an endemic plague focus in Madagascar: implications for rodent surveillance and management

PLOS ONE

Dear Dr. Brouat,

Thank you for submitting your manuscript to PLOS ONE. After careful consideration, we feel that it has merit but does not fully meet PLOS ONE’s publication criteria as it currently stands. Therefore, we invite you to submit a revised version of the manuscript that addresses the points raised during the review process.

Please submit your revised manuscript by Jan 12 2026 11:59PM. If you will need more time than this to complete your revisions, please reply to this message or contact the journal office at plosone@plos.org. When you're ready to submit your revision, log on to https://www.editorialmanager.com/pone/ and select the 'Submissions Needing Revision' folder to locate your manuscript file.

Please include the following items when submitting your revised manuscript:

- A rebuttal letter that responds to each point raised by the academic editor and reviewer(s). You should upload this letter as a separate file labeled 'Response to Reviewers'.
- A marked-up copy of your manuscript that highlights changes made to the original version. You should upload this as a separate file labeled 'Revised Manuscript with Track Changes'.
- An unmarked version of your revised paper without tracked changes. You should upload this as a separate file labeled 'Manuscript'.

If you would like to make changes to your financial disclosure, please include your updated statement in your cover letter. Guidelines for resubmitting your figure files are available below the reviewer comments at the end of this letter.

If applicable, we recommend that you deposit your laboratory protocols in protocols.io to enhance the reproducibility of your results. Protocols.io assigns your protocol its own identifier (DOI) so that it can be cited independently in the future. For instructions see: https://journals.plos.org/plosone/s/submission-guidelines#loc-laboratory-protocols. Additionally, PLOS ONE offers an option for publishing peer-reviewed Lab Protocol articles, which describe protocols hosted on protocols.io. Read more information on sharing protocols at https://plos.org/protocols?utm_medium=editorial-email&utm_source=authorletters&utm_campaign=protocols.

We look forward to receiving your revised manuscript.

Kind regards,

Tzen-Yuh Chiang

Academic Editor

PLOS ONE

Journal Requirements:

When submitting your revision, we need you to address these additional requirements.

1.Please ensure that your manuscript meets PLOS ONE's style requirements, including those for file naming. The PLOS ONE style templates can be found at

https://journals.plos.org/plosone/s/file?id=wjVg/PLOSOne_formatting_sample_main_body.pdf and

https://journals.plos.org/plosone/s/file?id=ba62/PLOSOne_formatting_sample_title_authors_affiliations.pdf

2. In your Methods section, please provide additional information regarding the permits you obtained for the work. Please ensure you have included the full name of the authority that approved the field site access and, if no permits were required, a brief statement explaining why.

3. Thank you for stating the following financial disclosure:

“Financial support to conduct fieldworks and laboratory analyses (from 2018 to 2021) was graciously provided by the Directorate General for International Relations and Strategy (DGRIS) with scientific support of IRBA (Institut de Recherche Biomédicale des forces Armées, France), within the framework of French MoD’s involvement in G7 Global partnership. The Agence Française d’Expertise Technique Internationale (AFETI) ensured the project’s financial execution (ref: 2018-SB-024-018 SSE OCO49-PMG7- IPMMADAGASCAR) during this period and contributed to the implementation of cooperation actions under the control of the DGRIS.”

Please state what role the funders took in the study.  If the funders had no role, please state: "The funders had no role in study design, data collection and analysis, decision to publish, or preparation of the manuscript."

If this statement is not correct you must amend it as needed.

Please include this amended Role of Funder statement in your cover letter; we will change the online submission form on your behalf.

4. Please note that your Data Availability Statement is currently missing the repository name and/or the DOI/accession number of each dataset OR a direct link to access each database. If your manuscript is accepted for publication, you will be asked to provide these details on a very short timeline. We therefore suggest that you provide this information now, though we will not hold up the peer review process if you are unable.

5. We note that Figure 1 in your submission contain map/satellite images which may be copyrighted. All PLOS content is published under the Creative Commons Attribution License (CC BY 4.0), which means that the manuscript, images, and Supporting Information files will be freely available online, and any third party is permitted to access, download, copy, distribute, and use these materials in any way, even commercially, with proper attribution. For these reasons, we cannot publish previously copyrighted maps or satellite images created using proprietary data, such as Google software (Google Maps, Street View, and Earth). For more information, see our copyright guidelines: http://journals.plos.org/plosone/s/licenses-and-copyright.

We require you to either (1) present written permission from the copyright holder to publish these figures specifically under the CC BY 4.0 license, or (2) remove the figures from your submission:

1. You may seek permission from the original copyright holder of Figure 1 to publish the content specifically under the CC BY 4.0 license.

We recommend that you contact the original copyright holder with the Content Permission Form (http://journals.plos.org/plosone/s/file?id=7c09/content-permission-form.pdf) and the following text:

“I request permission for the open-access journal PLOS ONE to publish XXX under the Creative Commons Attribution License (CCAL) CC BY 4.0 (http://creativecommons.org/licenses/by/4.0/). Please be aware that this license allows unrestricted use and distribution, even commercially, by third parties. Please reply and provide explicit written permission to publish XXX under a CC BY license and complete the attached form.”

Please upload the completed Content Permission Form or other proof of granted permissions as an "Other" file with your submission.

In the figure caption of the copyrighted figure, please include the following text: “Reprinted from [ref] under a CC BY license, with permission from [name of publisher], original copyright [original copyright year].”

2. If you are unable to obtain permission from the original copyright holder to publish these figures under the CC BY 4.0 license or if the copyright holder’s requirements are incompatible with the CC BY 4.0 license, please either i) remove the figure or ii) supply a replacement figure that complies with the CC BY 4.0 license. Please check copyright information on all replacement figures and update the figure caption with source information. If applicable, please specify in the figure caption text when a figure is similar but not identical to the original image and is therefore for illustrative purposes only.

The following resources for replacing copyrighted map figures may be helpful:

USGS National Map Viewer (public domain): http://viewer.nationalmap.gov/viewer/

The Gateway to Astronaut Photography of Earth (public domain): http://eol.jsc.nasa.gov/sseop/clickmap/

Maps at the CIA (public domain): https://www.cia.gov/library/publications/the-world-factbook/index.html and https://www.cia.gov/library/publications/cia-maps-publications/index.html

NASA Earth Observatory (public domain): http://earthobservatory.nasa.gov/

Landsat: http://landsat.visibleearth.nasa.gov/

USGS EROS (Earth Resources Observatory and Science (EROS) Center) (public domain): http://eros.usgs.gov/#

Natural Earth (public domain): http://www.naturalearthdata.com/

6. If the reviewer comments include a recommendation to cite specific previously published works, please review and evaluate these publications to determine whether they are relevant and should be cited. There is no requirement to cite these works unless the editor has indicated otherwise.

[Note: HTML markup is below. Please do not edit.]

Reviewers' comments:

Reviewer's Responses to Questions

**Comments to the Author**

1. Is the manuscript technically sound, and do the data support the conclusions?

The manuscript must describe a technically sound piece of scientific research with data that supports the conclusions. Experiments must have been conducted rigorously, with appropriate controls, replication, and sample sizes. The conclusions must be drawn appropriately based on the data presented.

Reviewer #1: Yes

**********

2. Has the statistical analysis been performed appropriately and rigorously?

Reviewer #1: Yes

**********

3. Have the authors made all data underlying the findings in their manuscript fully available?

The PLOS Data policy requires authors to make all data underlying the findings described in their manuscript fully available without restriction, with rare exception (please refer to the Data Availability Statement in the manuscript PDF file). The data should be provided as part of the manuscript or its supporting information, or deposited to a public repository. For example, in addition to summary statistics, the data points behind means, medians and variance measures should be available. If there are restrictions on publicly sharing data—e.g. participant privacy or use of data from a third party—those must be specified.

Reviewer #1: No

**********

4. Is the manuscript presented in an intelligible fashion and written in standard English?

PLOS ONE does not copyedit accepted manuscripts, so the language in submitted articles must be clear, correct, and unambiguous. Any typographical or grammatical errors should be corrected at revision, so please note any specific errors here.

Reviewer #1: Yes

**********

5. Review Comments to the Author

Please use the space provided to explain your answers to the questions above. You may also include additional comments for the author, including concerns about dual publication, research ethics, or publication ethics. (Please upload your review as an attachment if it exceeds 20,000 characters)

Reviewer #1: The manuscript aimed to report the genetic structure of the black rat in plague focus district of Madagascar.

Comment (to the journal?): Line numbers should be added.

‘microsatellite’ in the keywords should be in plural with regards to the plurality of biomarkers, or ‘microsatellite markers’

Abstract:

As you have focused the paper on data from two trapping sessions, one in March-May 2019 and another in March-May 2020, is ‘temporal structure’ still relevant? Does significant change of genetic structure occur in such duration?

Change ‘Our results show’ to ‘…. showed’

‘… combining flea control within houses’ may be ‘… flea control inside houses’?

Introduction:

‘… in close contact with people’ => ‘… with humans’?

‘central highlands’ to ‘Central Highlands’

‘above 800 m high’ to ‘at elevation above 800m’

‘to the relief in the central highlands’ => ‘…. Central Highlands’

‘inside village houses’ => ‘inside houses’

Materials and methods :

‘central Highlands (mean altitude of 1,190 m)’ => ‘Central Highlands…’

‘altitude’ => ‘elevation’

‘namely: Ambohitromby’ => ‘namely Ambohitromby’

‘Hills slopes’ => ‘Hill slopes’

Trapping sessions: as you are focused on two trapping sessions, it may not be necessary to mention ‘Six small TS…’

Specify meaning of ‘night-traps’ (calculation) to ease understanding of 3,888 going from 18 houses during three nights.

Is there any particular reason why trapping efforts were different between habitats? 18 houses (36 traps?) against 84 traps outside villages.

‘Species, sex, external measurements (head-body length, tail length, ear length and hind foot length, in mm) and weight (in g) were recorded on each individual.’ You can arrange sentence to say that body mensuration (or external measurement) allowed identifying rodent at species level.

‘Kidney of each individual was sampled then stored in 95° ethanol for subsequent molecular investigation purpose. Furthermore, blood was collected by cardiac puncture and centrifuged to obtain sera samples for serological analyses.’ I think blood sampling goes before kidney sampling?

‘For each individual, total DNA was extracted from kidney using QIAamp DNA Mini Kit (Qiagen, Hilden, Germany) as recommended by the supplier’. Manufacturer instead of supplier.

A table showing microsatellite marker features would be helpful. How 18 markers were pooled during multiplex reactions? What is the expected amplicon size per locus? Here there are 18 markers whereas in the abstract, there are 17. Also, can authors explain the use of markers developed for brown rat to test black rat? Isn’t it required to use species-specific microsat markers?

‘Detection was conducted using an ABI 3130xl Genetic Analyzers (Applied Biosystems, Foster City, CA, USA) and genotypes were red, analysed…’. Detection of ….? Read instead of red?

‘Each group of samples from a given habitat × locality × session was considered as a distinct subpopulation in the subsequent analyses.’ You can change to ‘Individuals belonging to a given habitat (inside house or outside village), locality (the same site?), and sampling session (2019 or 2020) were considered as a distinct subpopulation in the subsequent analyses’

‘Nei’s unbiased genetic diversity (HE, [28]) and FIS’. Change to ‘………….. and the inbreeding coefficient FIS’.

Can authors spell out LD methods?

For IBD analysis, please specify what is considered as subpopulation? Still pop from a habitat?

‘Rats captured inside houses during March-May 2020 in Antakavana and Ambolotarakely were excluded from the dataset due to low sample sizes (n = 6 and n = 7, respectively) (Fig 1).’ You can also cite Table 1 in addition to Fig1.

Table 1:

HS meaning?

Results:

‘STRUCTURE was used to investigate whether rat populations where spatially structured in Ankazobe district.’ Were instead of where.

Discussion:

‘During our 2 year-long survey’. If I’m not wrong, samplings were performed once per year during two years.

As the genetic diversity varied according to habitat (higher outside village), how is the connection/gene exchanges between populations from inside houses with those from outside? In the bibliography, rat would move from field to inside houses following harvest.

‘the observation of different ectoparasites on rats’ change to ‘… different fleas…’

**********

6. PLOS authors have the option to publish the peer review history of their article (what does this mean?). If published, this will include your full peer review and any attached files.

If you choose “no”, your identity will remain anonymous but your review may still be made public.

**Do you want your identity to be public for this peer review?**  For information about this choice, including consent withdrawal, please see our Privacy Policy.

Reviewer #1: No

**********

[NOTE: If reviewer comments were submitted as an attachment file, they will be attached to this email and accessible via the submission site. Please log into your account, locate the manuscript record, and check for the action link "View Attachments". If this link does not appear, there are no attachment files.]

To ensure your figures meet our technical requirements, please review our figure guidelines: https://journals.plos.org/plosone/s/figures

You may also use PLOS’s free figure tool, NAAS, to help you prepare publication quality figures: https://journals.plos.org/plosone/s/figures#loc-tools-for-figure-preparation.

NAAS will assess whether your figures meet our technical requirements by comparing each figure against our figure specifications.

# Author comment: Author response to Decision Letter 1

**DOI:** 10.1371/journal.pone.0334819.r002

16 Dec 2025

We thank the reviewers for their constructive comments. We carefully addressed all points raised. Detailed responses to each comment are provided below:

Reviewer comment: ‘microsatellite’ in the keywords should be in plural with regards to the plurality of biomarkers, or ‘microsatellite markers.

Author: we have corrected the keyword to “microsatellite markers” (l. 40).

Reviewer comment: Abstract: As you have focused the paper on data from two trapping sessions, one in March-May 2019 and another in March-May 2020, is ‘temporal structure’ still relevant? Does significant change of genetic structure occur in such duration?

Author: we agree that the terms “temporal structure” may be misleading. We modified the sentence as: “this study aims at investigating the genetic structure of R. rattus populations at a fine geographical scale and across two different years” (l. 22-23).

Reviewer comment: Change ‘Our results show’ to ‘…. showed’.

Author: The correction was made in the revised version (l. 27).

Reviewer comment: ‘… combining flea control within houses’ may be ‘… flea control inside houses’?

Author: the correction was made in the revised version (l. 36).

Reviewer comment: Introduction: ‘… in close contact with people’ => ‘… with humans’?

Author: the correction was made in the revised version (l. 49).

Reviewer comment: ‘central highlands’ to ‘Central Highlands’.

Author: the correction was made in the revised version (l. 55-56).

Reviewer comment: ‘above 800 m high’ to ‘at elevation above 800m’.

Author: the correction was made in the revised version (l. 56).

Reviewer comment: ‘to the relief in the central highlands’ => ‘…. Central Highlands’.

Author: the correction was made in the revised version (l. 70-71).

Reviewer comment: ‘inside village houses’ => ‘inside houses’.

Author: the correction was made in the revised version (l. 79).

Reviewer comment: Materials and methods: ‘central Highlands (mean altitude of 1,190 m)’ => ‘Central Highlands…’

Author: the correction was made in the revised version (l. 85).

Reviewer comment: ‘altitude’ => ‘elevation’.

Author: the correction was made in the revised version (l. 85).

Reviewer comment: ‘namely: Ambohitromby’ => ‘namely Ambohitromby’.

Author: the correction was made in the revised version (l. 86).

Reviewer comment: ‘Hills slopes’ => ‘Hill slopes’.

Author: the correction was made in the revised version (l. 90).

Reviewer comment: Trapping sessions: as you are focused on two trapping sessions, it may not be necessary to mention ‘Six small TS…’

Author: this part of the text was reworded (l. 99-104), in order to focus on the two trapping sessions that were considered.

Reviewer comment: Specify meaning of ‘night-traps’ (calculation) to ease understanding of 3,888 going from 18 houses during three nights.

Author: we agree that the total number of 3,888 night-traps was confusing, especially since it referred to the six initial trapping sessions among which only two were considered in this manuscript. We removed indication of the total number of night-traps and clarified the trapping design by specifying the number of traps, houses, and nights only (l. 108-110).

Reviewer comment: Is there any particular reason why trapping efforts were different between habitats? 18 houses (36 traps?) against 84 traps outside villages.

Author: we thank the reviewer for this comment. The difference in trapping effort between indoor habitats and outside villages was in part due to logistical and ethical constraints: indoor trapping could only be conducted in houses where the owner or tenant had provided verbal consent. Importantly, our objective was not to compare trap success between indoor and outside village habitats, but rather to collect sufficient numbers of individuals from each habitat in each locality to perform the population genetic analyses. For this reason, we believe that the difference in trapping effort has a limited impact on the interpretation of our results.

Reviewer comment: ‘Species, sex, external measurements (head-body length, tail length, ear length and hind foot length, in mm) and weight (in g) were recorded on each individual.’ You can arrange sentence to say that body mensuration (or external measurement) allowed identifying rodent at species level.

Author: The sentence was modified as suggested by the reviewer (l. 115-120).

Reviewer comment: Kidney of each individual was sampled then stored in 95° ethanol for subsequent molecular investigation purpose. Furthermore, blood was collected by cardiac puncture and centrifuged to obtain sera samples for serological analyses. I think blood sampling goes before kidney sampling?

Author: the sentences have been modified to indicate that blood was collected before to kidney sampling (l. 120-124).

Reviewer comment: For each individual, total DNA was extracted from kidney using QIAamp DNA Mini Kit (Qiagen, Hilden, Germany) as recommended by the supplier. Manufacturer instead of supplier.

Author: the correction was made in the revised version (l. 132-133).

Reviewer comment: A table showing microsatellite marker features would be helpful. How 18 markers were pooled during multiplex reactions? What is the expected amplicon size per locus? Here there are 18 markers whereas in the abstract, there are 17. Also, can authors explain the use of markers developed for brown rat to test black rat? Isn’t it required to use species-specific microsat markers?

Author: a table showing microsatellite marker features, expected amplicon size per locus, and marker ID pooling information was provided as Supplementary Material in the revised version (S1 Table) (l. 137).

Individuals were genotyped using 18 markers, but one of them was excluded from the analysis given the number of null genotypes. We corrected the abstract for more clarity.

Regarding the use of markers developed for brown rat (Rattus norvegicus), these loci have been successfully applied to black rat (R. rattus) in previous studies, showing reliable amplification and sufficient polymorphism (Brouat et al., 2013; Brouat et al., 2014; Berthier et al., 2016; Badou et al., 2023).

Reviewer comment: ‘Detection was conducted using an ABI 3130xl Genetic Analyzers (Applied Biosystems, Foster City, CA, USA) and genotypes were red, analysed…’. Detection of ….? Read instead of red?

Author: the sentence was modified as following: “Genotyping was performed with an ABI 3130xl Genetic Analyzers (Applied Biosystems, Foster City, CA, USA) and microsatellite profiles were read, analysed and eye-cleaned under GeneMapper v.6 software.”(l. 139-141).

Reviewer comment: ‘Each group of samples from a given habitat × locality × session was considered as a distinct subpopulation in the subsequent analyses.’ You can change to ‘Individuals belonging to a given habitat (inside house or outside village), locality (the same site?), and sampling session (2019 or 2020) were considered as a distinct subpopulation in the subsequent analyses’

Author: the sentence was modified as suggested in the revised version (l. 144-146).

Reviewer comment: ‘Nei’s unbiased genetic diversity (HE, [28]) and FIS’. Change to ‘………….. and the inbreeding coefficient FIS’.

Author: the correction was made in the revised version (l. 154).

Reviewer comment: Can authors spell out LD methods?

Author: the correction was made in the revised version (l. 155-156).

Reviewer comment: For IBD analysis, please specify what is considered as subpopulation? Still pop from a habitat?

Author: yes, subpopulation is used as described above in the manuscript, and as clarified by the reviewer (see above).

Reviewer comment: ‘Rats captured inside houses during March-May 2020 in Antakavana and Ambolotarakely were excluded from the dataset due to low sample sizes (n = 6 and n = 7, respectively) (Fig 1).’ You can also cite Table 1 in addition to Fig1.

Author: citation of Table 1 was added in the revised version (l. 217).

Reviewer comment: Table 1: HS meaning?

Author: “HS in Table 1 was corrected to HE (expected heterozygosity).

Reviewer comment: Results: ‘STRUCTURE was used to investigate whether rat populations where spatially structured in Ankazobe district.’ Were instead of where.

Author: the correction was made in the revised version (l. 270).

Reviewer comment: Discussion: ‘During our 2 year-long survey’. If I’m not wrong, samplings were performed once per year during two years.

Author: we clarified the sentence to indicate that the two-year surveillance study, including the two trapping sessions analyzed here, revealed low seroprevalence of Y. pestis in small mammals across the six localities (Parany et al., 2025) (l. 299-302).

Reviewer comment: As the genetic diversity varied according to habitat (higher outside village), how is the connection/gene exchanges between populations from inside houses with those from outside? In the bibliography, rat would move from field to inside houses following harvest.

Author: our results indicated low but significant differentiation between rat populations inside houses and outside villages. This differentiation suggests limited effective dispersal among habitats, but movements of individuals may occur regularly. The observed difference in genetic diversity could be explained by differences in population size.

Reviewer comment: ‘the observation of different ectoparasites on rats’ change to ‘… different fleas…’

Author: the correction was made in the revised version (l. 344).

Attachment Submitted filename: Response to Reviewers.docx

# Peer review: Decision Letter 1

**DOI:** 10.1371/journal.pone.0334819.r003

**Authors:** Tzen-Yuh Chiang, Tzen-Yuh Chiang

30 Jan 2026

PONE-D-25-53545R1

Genetic structure of *Rattus rattus*  populations in an endemic plague focus in Madagascar: implications for rodent surveillance and management

PLOS One

Dear Dr. Brouat,

Thank you for submitting your manuscript to PLOS ONE. After careful consideration, we feel that it has merit but does not fully meet PLOS ONE’s publication criteria as it currently stands. Therefore, we invite you to submit a revised version of the manuscript that addresses the points raised during the review process.

Please submit your revised manuscript by Mar 16 2026 11:59PM. If you will need more time than this to complete your revisions, please reply to this message or contact the journal office at plosone@plos.org. When you're ready to submit your revision, log on to https://www.editorialmanager.com/pone/ and select the 'Submissions Needing Revision' folder to locate your manuscript file.

Please include the following items when submitting your revised manuscript:

- A letter that responds to each point raised by the academic editor and reviewer(s). You should upload this letter as a separate file labeled 'Response to Reviewers'.
- A marked-up copy of your manuscript that highlights changes made to the original version. You should upload this as a separate file labeled 'Revised Manuscript with Track Changes'.
- An unmarked version of your revised paper without tracked changes. You should upload this as a separate file labeled 'Manuscript'.

-->If you would like to make changes to your financial disclosure, please include your updated statement in your cover letter. Guidelines for resubmitting your figure files are available below the reviewer comments at the end of this letter.

If applicable, we recommend that you deposit your laboratory protocols in protocols.io to enhance the reproducibility of your results. Protocols.io assigns your protocol its own identifier (DOI) so that it can be cited independently in the future. For instructions see: https://journals.plos.org/plosone/s/submission-guidelines#loc-laboratory-protocols. Additionally, PLOS ONE offers an option for publishing peer-reviewed Lab Protocol articles, which describe protocols hosted on protocols.io. Read more information on sharing protocols at https://plos.org/protocols?utm_medium=editorial-email&utm_source=authorletters&utm_campaign=protocols.

We look forward to receiving your revised manuscript.

Kind regards,

Tzen-Yuh Chiang

Academic Editor

PLOS One

Journal Requirements:

1. If the reviewer comments include a recommendation to cite specific previously published works, please review and evaluate these publications to determine whether they are relevant and should be cited. There is no requirement to cite these works unless the editor has indicated otherwise.

2. Please review your reference list to ensure that it is complete and correct. If you have cited papers that have been retracted, please include the rationale for doing so in the manuscript text, or remove these references and replace them with relevant current references. Any changes to the reference list should be mentioned in the rebuttal letter that accompanies your revised manuscript. If you need to cite a retracted article, indicate the article’s retracted status in the References list and also include a citation and full reference for the retraction notice.

[Note: HTML markup is below. Please do not edit.]

Reviewers' comments:

Reviewer's Responses to Questions

**Comments to the Author**

1. If the authors have adequately addressed your comments raised in a previous round of review and you feel that this manuscript is now acceptable for publication, you may indicate that here to bypass the “Comments to the Author” section, enter your conflict of interest statement in the “Confidential to Editor” section, and submit your "Accept" recommendation.

Reviewer #1: All comments have been addressed

Reviewer #2: All comments have been addressed

Reviewer #3: (No Response)

**********

2. Is the manuscript technically sound, and do the data support the conclusions?

The manuscript must describe a technically sound piece of scientific research with data that supports the conclusions. Experiments must have been conducted rigorously, with appropriate controls, replication, and sample sizes. The conclusions must be drawn appropriately based on the data presented.

Reviewer #1: Yes

Reviewer #2: Yes

Reviewer #3: Yes

**********

3. Has the statistical analysis been performed appropriately and rigorously?

Reviewer #1: Yes

Reviewer #2: Yes

Reviewer #3: Yes

**********

4. Have the authors made all data underlying the findings in their manuscript fully available?

The PLOS Data policy requires authors to make all data underlying the findings described in their manuscript fully available without restriction, with rare exception (please refer to the Data Availability Statement in the manuscript PDF file). The data should be provided as part of the manuscript or its supporting information, or deposited to a public repository. For example, in addition to summary statistics, the data points behind means, medians and variance measures should be available. If there are restrictions on publicly sharing data—e.g. participant privacy or use of data from a third party—those must be specified.

Reviewer #1: Yes

Reviewer #2: Yes

Reviewer #3: No

**********

5. Is the manuscript presented in an intelligible fashion and written in standard English?

PLOS ONE does not copyedit accepted manuscripts, so the language in submitted articles must be clear, correct, and unambiguous. Any typographical or grammatical errors should be corrected at revision, so please note any specific errors here.

Reviewer #1: Yes

Reviewer #2: Yes

Reviewer #3: Yes

**********

6. Review Comments to the Author

Please use the space provided to explain your answers to the questions above. You may also include additional comments for the author, including concerns about dual publication, research ethics, or publication ethics. (Please upload your review as an attachment if it exceeds 20,000 characters)

Reviewer #1: My previous comments are addressed by authors. The revised version was highly improved.

I just have one comment/question:

Lines 125-126 : eight or seven markers ?

Reviewer #2: This is my first review of this manuscript. I understand it has undergone previous review, and I acknowledge the work done to reach this stage. The manuscript is clear and the results are relevant for pest control.

However, my main question relates to the clarity and justification of the research objective concerning the temporal genetic structure between the two sampling years, which are only one year apart. The introduction does not sufficiently establish why investigating genetic changes from one year to another (2019 vs 2020) is a worthwhile question for this system.

See specifics below:

Ln68: Could you please elaborate on these previous results, particularly their spatial extent? While the importance of the spatial scale is clear, the relevance of the temporal scale is less obvious. What is the generation time of the rat population? Is the consideration of time primarily due to seasonal variations in rat abundance? Or, is it because rats are routinely exterminated within houses each year, leading us to expect that rat colonization events are an annual occurrence? Please make clear justification of the objectives regarding the temporal question. Relying on the reader to piece it together from the introduction and methods reduces the text's clarity and impact.

Ln97-99: In terms of distance, what average distance was from inside the house’s vs outside of the village? The distance between villages is provided, but which were the min. and max. distances between the two sampling habitats? This is relevant to interpreting the results.

Table 1. Session S1 and S2 relate to the two sampling years? Why not instead to put the years S2019, S2020

L223: among sampling sessions

Ln265-268: I don’t have clear why genetic structure was analyzed separately for inside and outside villages?

Ln317: this information should be in the introduction, I have still no clarity of why comparing two sampling years.

Ln359-362: Expand further the management recommendation provided; I don’t see much difference between what is being done and what is being recommended. Again, the initial question regarding year to year differences is not clear, and not linked to a specific biology of the species or related to a particular pest control management.

Reviewer #3: Body of work presents technically sound original research.

Would be nice to see comparisons made to somewhat similar work published in the Canadian Journal of Zoology, 2007. This work feels like a scale up of what was done there?

Transparency is important and I feel the authors should share how the analyses were done, instills confidence that the work was done as written and imparts knowledge to others.

Microsatellite genotyping section - please indicate temporal distinction of the samples genotyped. Also, based on the rates of evolution for some molecular marker's, I am not sure one can expect to see temporal difference with microsats over a year's time, even if you managed to catch the same individual?

Data analyses section - please indicate what tools what were used at the very start or first few lines of the paragraph. Also, may just mention 'R v 4.4.2' instead of 'R software version....'.

Not sure non-parametric tests will give anything remotely definitive on plague leaving a trace on genetic diversity? unless the objective is to just give an association? somewhat superficial relationship? Certain you are aware non-parametric tests don't provide a cause-effect relationship.

Line 183: did not see mention of STRUCTURE and DAPC analyses being used to test temporal variation? unless I missed whether this was stated?

In my experience STRUCTURE and DAPC rarely give you similar answers, always a '1 K' margin of error...of course this is dependent on inherent differences in the marker of choice and algorithmic biases...please review all mentions of DAPC as confirmatory analyses and suggest that changes to them being secondary analyses.

Great work.

**********

7. PLOS authors have the option to publish the peer review history of their article (what does this mean?). If published, this will include your full peer review and any attached files.

If you choose “no”, your identity will remain anonymous but your review may still be made public.

**Do you want your identity to be public for this peer review?**  For information about this choice, including consent withdrawal, please see our Privacy Policy.

Reviewer #1: No

Reviewer #2: No

Reviewer #3: No

**********

[NOTE: If reviewer comments were submitted as an attachment file, they will be attached to this email and accessible via the submission site. Please log into your account, locate the manuscript record, and check for the action link "View Attachments". If this link does not appear, there are no attachment files.]

To ensure your figures meet our technical requirements, please review our figure guidelines: https://journals.plos.org/plosone/s/figures

You may also use PLOS’s free figure tool, NAAS, to help you prepare publication quality figures: https://journals.plos.org/plosone/s/figures#loc-tools-for-figure-preparation.

NAAS will assess whether your figures meet our technical requirements by comparing each figure against our figure specifications.

# Author comment: Author response to Decision Letter 2

**DOI:** 10.1371/journal.pone.0334819.r004

24 Feb 2026

We thank the reviewers for their constructive comments. We carefully addressed all points raised. Detailed responses to each comment are provided below:

Reviewer #1: My previous comments are addressed by authors. The revised version was highly improved.

I just have one comment/question:

Lines 125-126: eight or seven markers?

Author: The number of microsatellite markers developed for R. norvegicus is eight: the D5Rat83 was added in the marker list in the revised version (l. 159 in the revised version with tracked changes).

Reviewer #2: This is my first review of this manuscript. I understand it has undergone previous review, and I acknowledge the work done to reach this stage. The manuscript is clear and the results are relevant for pest control.

However, my main question relates to the clarity and justification of the research objective concerning the temporal genetic structure between the two sampling years, which are only one year apart. The introduction does not sufficiently establish why investigating genetic changes from one year to another (2019 vs 2020) is a worthwhile question for this system.

Author: we thank the reviewer for this comment and agree that the objective required clarification. We have revised the Introduction accordingly (l. 83-97 and 101-108). Our objective in comparing 2019 and 2020 samples was to assess the short-term temporal stability of fine-scale genetic structure, rather than to investigate long-term evolutionary change. Black rats have short generation times (about 6 months) and high reproductive rates, so a one-year interval may encompass different generations. In addition, rat populations in the Central Highlands undergo marked seasonal fluctuations in abundance and may be affected by plague-induced mortality and human-mediated dispersal among villages. These factors could theoretically lead to rapid shifts in allele frequencies at a local scale. Testing temporal stability over one year is therefore important to determine whether the observed spatial genetic structure reflects persistent local populations or transient demographic dynamics. Demonstrating stability (or instability) directly informs the interpretation of gene flow and connectivity among villages, and is thus important regarding the implementation of plague control measures.

Reviewer #2: See specifics below:

Ln68: Could you please elaborate on these previous results, particularly their spatial extent? While the importance of the spatial scale is clear, the relevance of the temporal scale is less obvious. What is the generation time of the rat population? Is the consideration of time primarily due to seasonal variations in rat abundance? Or, is it because rats are routinely exterminated within houses each year, leading us to expect that rat colonization events are an annual occurrence? Please make clear justification of the objectives regarding the temporal question. Relying on the reader to piece it together from the introduction and methods reduces the text's clarity and impact.

Author: we agree with the reviewer, and have elaborate on previous results in this part (l. 83-97). As explained just above, we have also clarified why we aim to describe the temporal genetic structure (l. 101-108).

Reviewer #2: Ln97-99: In terms of distance, what average distance was from inside the house’s vs outside of the village? The distance between villages is provided, but which were the min. and max. distances between the two sampling habitats? This is relevant to interpreting the results.

Author: within localities, the minimum distance between sampling habitats (inside houses vs. outside the village) was 11.4 m, the maximum distance was 234 m, and the mean distance was 106 m. These distances were calculated using GPS coordinates with the R package geosphere, representing straight-line distances. The real paths used by black rats may differ due to landscape features.

This information has been added in the revised version (l. 128-129).

Reviewer #2: Table 1. Session S1 and S2 relate to the two sampling years? Why not instead to put the years S2019, S2020

Author: the labels “S1” and “S2” were replaced with “S.2019” and “S.2020” in the Table 1 and throughout the revised manuscript and in figures.

Reviewer #2: L223: among sampling sessions

Author: the correction was made in the revised version (l. 263).

Reviewer #2: Ln265-268: I don’t have clear why genetic structure was analyzed separately for inside and outside villages?

Author: we thank the reviewer for this comment. We analyzed genetic structure separately for individuals sampled inside houses and outside villages because these two habitats represent distinct ecological contexts that may influence rat population dynamics and connectivity. Indeed, rats living inside houses benefit from more stable food resources and shelter, and may reproduce continuously. As a result, indoor populations may function as relatively discrete demographic units, potentially more resistant to immigration from surrounding outdoor populations. In contrast, rats living outside villages reproduce seasonally, and may experience increased dispersal among populations during periods of low density, likely associated with greater spatial connectivity, thereby forming a more continuous population across the landscape. We therefore hypothesized that genetic structuring might be more detectable among house-dwelling subpopulations than among outdoor individuals. Separate analyses were conducted to test this ecological hypothesis and to assess whether habitat type influences fine-scale genetic patterns relevant for plague control strategies. We have clearly stated this hypothesis in the Material and method (l. 221-225).

Reviewer #2: Ln317: this information should be in the introduction, I have still no clarity of why comparing two sampling years.

Author: information about generation time was added in the introduction section of the revised version (l. 93-94), and the objective to compare two sampling years has been made more explicit (see above).

Reviewer #2: Ln359-362: Expand further the management recommendation provided; I don’t see much difference between what is being done and what is being recommended. Again, the initial question regarding year-to-year differences is not clear, and not linked to a specific biology of the species or related to a particular pest control management.

Author: as clarified in the introduction of the revised version (l. 83-97 and l. 101-108) and in the last paragraph of the discussion related to management recommendation (l. 400-414), the temporal analysis was designed to clarify the biological meaning of the observed spatial genetic structure. Specifically, comparing two consecutive years allowed us to determine whether the spatial differentiation detected among villages reflected stable population structuring or transient patterns driven by rapid turnover and recolonization dynamics. The absence of significant temporal genetic differentiation indicates that allele frequencies and clustering patterns are stable over time, supporting the interpretation that rat populations at the district scale form a persistent and genetically connected system rather than ephemeral, independently fluctuating units. This temporal stability therefore strengthens the inference drawn from spatial analyses, namely that villages do not constitute isolated management units. We hope that it is clearer now in the revised version.

Reviewer #3: Body of work presents technically sound original research.

Would be nice to see comparisons made to somewhat similar work published in the Canadian Journal of Zoology, 2007. This work feels like a scale up of what was done there?

Author: we thank the reviewer for this suggestion. The comparison with Gilabert et al., 2007 is now explicitly made in the revised version (l. 373-377).

Reviewer #3: Transparency is important and I feel the authors should share how the analyses were done, instills confidence that the work was done as written and imparts knowledge to others.

Author: we thank the reviewer for emphasizing the importance of transparency. All analytical procedures are described in detail in the methods section to ensure reproducibility and clarity. In addition, the genotype dataset has been made publicly available in the Zenodo repository at the following link: https://doi.org/10.5281/zenodo.17810727 and details of microsatellites markers and primer sequences with their fluorescent dyes, expected fragments and pooling group of markers for multiplex PCR are available in the supporting information.

Reviewer #3: Microsatellite genotyping section - please indicate temporal distinction of the samples genotyped. Also, based on the rates of evolution for some molecular marker's, I am not sure one can expect to see temporal difference with microsats over a year's time, even if you managed to catch the same individual?

Author: sample size per locality, habitat and session are specified in Table 1: we have clarified the reference to this table in the revised version, and added references to sampling sessions (l. 265-266). We agree that the rationale for comparing two temporally close sampling years was not sufficiently explained in the original version, a concern that was also raised by Reviewer 2. Regarding the evolution rate of microsatellites, previous studies already reported temporal variation in the genetic structure of black rat populations over short time intervals using microsatellites markers (for instance, see Berthier et al. 2006 in Mol. Ecol.; Kajdacsi et al. 2023 in Mol. Ecol.). We hope that the revised version and the detailed responses above satisfactorily address the reviewer’s comments.

Reviewer #3: Data analyses section - please indicate what tools what were used at the very start or first few lines of the paragraph. Also, may just mention 'R v 4.4.2' instead of 'R software version....'.

Author: as we used several software and tools, we propose to keep the mention of each software immediately after the corresponding test or analysis to avoid confusion. The R software version is specified in the revised version (l. 172).

Reviewer #3: Not sure non-parametric tests will give anything remotely definitive on plague leaving a trace on genetic diversity? unless the objective is to just give an association? somewhat superficial relationship? Certain you are aware non-parametric tests don't provide a cause-effect relationship.

Author: we thank the reviewer for this comment. Our objective in using these tests was solely to explore potential associations between the presence of plague and patterns of genetic diversity, rather than to infer a cause-effect relationship. The sentence was modified to clarify this point in the revised version (l. 182-187).

Reviewer #3: Line 183: did not see mention of STRUCTURE and DAPC analyses being used to test temporal variation? unless I missed whether this was stated?

Author: global STRUCTURE and DAPC analyses, including all sites, habitats, and trapping sessions, were indeed performed. These analyses revealed no signal for temporal genetic structure, and given these results, we did not conduct separate analyses by trapping session (S.2019 and S.2020). In addition, unlike for the spatial analyses, we did not formulate hypotheses that could explain temporal variation.

Reviewer #3: In my experience STRUCTURE and DAPC rarely give you similar answers, always a '1 K' margin of error...of course this is dependent on inherent differences in the marker of choice and algorithmic biases...please review all mentions of DAPC as confirmatory analyses and suggest that changes to them being secondary analyses.

Author: we have revised the manuscript to present DAPC analyses as a confirmatory analysis, individual-based approach to explore population structure (l. 215-216).

Attachment Submitted filename: Response to Reviewers_2.docx

# Peer review: Decision Letter 2

**DOI:** 10.1371/journal.pone.0334819.r005

**Authors:** Tzen-Yuh Chiang, Tzen-Yuh Chiang, Tzen-Yuh Chiang

1 Apr 2026

PONE-D-25-53545R2

Genetic structure of *Rattus rattus*  populations in an endemic plague focus in Madagascar: implications for rodent surveillance and management

PLOS One

Dear Dr. Brouat,

Thank you for submitting your manuscript to PLOS ONE. After careful consideration, we feel that it has merit but does not fully meet PLOS ONE’s publication criteria as it currently stands. Therefore, we invite you to submit a revised version of the manuscript that addresses the points raised during the review process.

Please submit your revised manuscript by May 16 2026 11:59PM. If you will need more time than this to complete your revisions, please reply to this message or contact the journal office at plosone@plos.org. When you're ready to submit your revision, log on to https://www.editorialmanager.com/pone/ and select the 'Submissions Needing Revision' folder to locate your manuscript file.

Please include the following items when submitting your revised manuscript:

- A letter that responds to each point raised by the academic editor and reviewer(s). You should upload this letter as a separate file labeled 'Response to Reviewers'.
- A marked-up copy of your manuscript that highlights changes made to the original version. You should upload this as a separate file labeled 'Revised Manuscript with Track Changes'.
- An unmarked version of your revised paper without tracked changes. You should upload this as a separate file labeled 'Manuscript'.

If you would like to make changes to your financial disclosure, please include your updated statement in your cover letter. Guidelines for resubmitting your figure files are available below the reviewer comments at the end of this letter.

If applicable, we recommend that you deposit your laboratory protocols in protocols.io to enhance the reproducibility of your results. Protocols.io assigns your protocol its own identifier (DOI) so that it can be cited independently in the future. For instructions see: https://journals.plos.org/plosone/s/submission-guidelines#loc-laboratory-protocols. Additionally, PLOS ONE offers an option for publishing peer-reviewed Lab Protocol articles, which describe protocols hosted on protocols.io. Read more information on sharing protocols at https://plos.org/protocols?utm_medium=editorial-email&utm_source=authorletters&utm_campaign=protocols.

As the corresponding author, your ORCID iD is verified in the submission system and will appear in the published article. PLOS supports the use of ORCID, and we encourage all coauthors to register for an ORCID iD and use it as well. Please encourage your coauthors to verify their ORCID iD within the submission system before final acceptance, as unverified ORCID iDs will not appear in the published article. *Only*  the individual author can complete the verification step; PLOS staff *cannot*  verify ORCID iDs on behalf of authors.

We look forward to receiving your revised manuscript.

Kind regards,

Tzen-Yuh Chiang

Academic Editor

PLOS One

Journal Requirements:

1. If the reviewer comments include a recommendation to cite specific previously published works, please review and evaluate these publications to determine whether they are relevant and should be cited. There is no requirement to cite these works unless the editor has indicated otherwise.

2. Please review your reference list to ensure that it is complete and correct. If you have cited papers that have been retracted, please include the rationale for doing so in the manuscript text, or remove these references and replace them with relevant current references. Any changes to the reference list should be mentioned in the rebuttal letter that accompanies your revised manuscript. If you need to cite a retracted article, indicate the article’s retracted status in the References list and also include a citation and full reference for the retraction notice.

[Note: HTML markup is below. Please do not edit.]

Reviewers' comments:

Reviewer's Responses to Questions

**Comments to the Author**

1. If the authors have adequately addressed your comments raised in a previous round of review and you feel that this manuscript is now acceptable for publication, you may indicate that here to bypass the “Comments to the Author” section, enter your conflict of interest statement in the “Confidential to Editor” section, and submit your "Accept" recommendation.

Reviewer #2: All comments have been addressed

Reviewer #4: (No Response)

Reviewer #5: (No Response)

Reviewer #6: (No Response)

**********

2. Is the manuscript technically sound, and do the data support the conclusions?

The manuscript must describe a technically sound piece of scientific research with data that supports the conclusions. Experiments must have been conducted rigorously, with appropriate controls, replication, and sample sizes. The conclusions must be drawn appropriately based on the data presented.

Reviewer #2: Yes

Reviewer #4: Yes

Reviewer #5: Partly

Reviewer #6: Yes

**********

3. Has the statistical analysis been performed appropriately and rigorously?

Reviewer #2: Yes

Reviewer #4: Yes

Reviewer #5: Yes

Reviewer #6: Yes

**********

4. Have the authors made all data underlying the findings in their manuscript fully available?

The PLOS Data policy requires authors to make all data underlying the findings described in their manuscript fully available without restriction, with rare exception (please refer to the Data Availability Statement in the manuscript PDF file). The data should be provided as part of the manuscript or its supporting information, or deposited to a public repository. For example, in addition to summary statistics, the data points behind means, medians and variance measures should be available. If there are restrictions on publicly sharing data—e.g. participant privacy or use of data from a third party—those must be specified.

Reviewer #2: Yes

Reviewer #4: Yes

Reviewer #5: Yes

Reviewer #6: No

**********

5. Is the manuscript presented in an intelligible fashion and written in standard English?

PLOS ONE does not copyedit accepted manuscripts, so the language in submitted articles must be clear, correct, and unambiguous. Any typographical or grammatical errors should be corrected at revision, so please note any specific errors here.

Reviewer #2: Yes

Reviewer #4: Yes

Reviewer #5: Yes

Reviewer #6: Yes

**********

6. Review Comments to the Author

Please use the space provided to explain your answers to the questions above. You may also include additional comments for the author, including concerns about dual publication, research ethics, or publication ethics. (Please upload your review as an attachment if it exceeds 20,000 characters)

Reviewer #2: I have read the revised version of this work and I found that the authors have addressed all my comments satisfactorily. I have no further comments.

Reviewer #4: The manuscript is clear and well structured, and the objectives, methods, and results are presented in a coherent and understandable manner. The authors have adequately addressed the issues raised during the review process, and the revised version meets the standards for publication. The study contributes relevant data to the understanding of rodent population dynamics in relation to disease surveillance.

I have no additional comments or concerns regarding research ethics or publication ethics. I support the publication of this manuscript.

Reviewer #5: This is my first revision of Parany et al. The study aims at quantifying genetic structure of rat populations occurring inside houses and outside houses across the Ankazobe district in the Central Highlands of Madagascar. This study is relevant in the context of plague outbreak, since rats are one of the major reservoir of plague spread. The authors used 17 microsatellites loci of >400 individuals sampled over two seasons (March-May 2019 and March-May 2020) to investigate genetic diversity, effective population size and genetic structure inside and outside houses, and across sampling seasons. They found higher genetic diversity and effective population size in the outside houses populations compared to the inside houses. There are also evidence of stronger genetic structure in the inside house populations. Overall, they conclude that the higher genetic diversity in the outside populations is likely due to the fact that there is higher dispersal among outside populations than inside populations. Higher dispersal in the outside populations suggest that rodent sureillance plans should not carry out on single subpopulations, but considering the whole network of rat connectivity.

The manuscipt is well written and clear. I think that they also present well the context. That being said I am not an expert on disease spread, plague and rat-plague co-dispersal so I cannot judge if the state of the art of the topic has been accurately presented.

I think that the authors have a quite unique dataset, although currently limited to microsatellites. I have some questions and suggestions that may help increase the impact of this work. I suggest the publication of this work upon minor revision.

General Comments

I might have missed it but it would have been interesting to see a quantitative comparison of plague seroprevalence between the inside vs outside houses rat populations. If the authors have enough data to quantfy such differences, it would also be interesting to assess whether differences in seroprevalence between inside and otside houses rats correlates with measures of genetic diversity (He and Fst) in the respective populations (inside and otside houses). Also, did the author computed seroprevalence per sampling seasons? Are there difference between those?

Also, I noticed that the authors reported genetic IBD statistics for the inside houses populations and overall sampling, but not among outside houses rat populations. Did I miss it or there is a specific reason for not doing that?

I think that in this context spatial analyses of genetic structure are quite relevant to understand how rat dispersal impact plague spread. I would suggest the authors to add few other spatial analyses which are related to genetic IBD but different, such as spatial autocorrelation or EEMS analysis (which measure rate of relative dispersal across a landscape using either microsatellite or SNP data) [https://github.com/dipetkov/eems].

Detailed Comments (numbering refers to the newest version of the manuscript I received)

Line 296: I think this sentence should be rephrased. The genetic IBD appears to be significant but negative in slope. I see what the authors meants, but I think it should be rephrased.

Line 297-299: what about genetic IBD for outside houses populations?

Line 282: I think it could be nice to better point to the south and northern populations in Fig. 3. I feel it is very difficult to see that at the moment.

Line 358-360: replace “larger sizes” with “larger effective population size”. Higher genetic diversity or Ne do not directly translate into higher census size, but it might be related to patterns of dispersal, higher in the outside houses than inside houses populations (as also the authors points out in the discussion).

Line 362-363: Could you measure relative abundance based on rat capture rate over the two sampling seasons and subpopulations?

Reviewer #6: Reviewer's report: Genetic structure of Rattus rattus populations in an endemic plague focus in

Madagascar: implications for rodent surveillance and management

This is my first review of the Parany et al. manuscript and I can see the authors have been thorough in responding to comments of all previous reviewers. This exploratory study provides an interesting comparison of genetic structure among rat subpopulations sampled from two environments: inside houses versus surrounding fields. The authors make key inferences regarding the rapid recolonization of rats, which is significant for plague control efforts in Madagascar.

Overall, the population genetic analyses are robust at both district-wide and local scales. However, some of the inferences are overstated, such as interpreting the genetic differentiation of Ankazobe (ANK) and Ambohitromby (AMB) as being a north-south pattern. ABK (a southern location) does not support a north-south hypothesis, based on the data presented. An alternative explanation that seems more plausible is given in the Discussion; namely, proximity to major urban centers which could potentially lead to gene flow into ANK and AMB. I recommend removing text related to a north-south pattern in the Discussion, and instead focus on the finding that ANK and AMB are members of a genetic group that is distinct from the other four locations.

Major Revisions:

1. Introduction: I saw the main hypothesis described in the Methods on lines 203-206. I think it would make the Introduction stronger if you also mentioned this hypothesis (briefly) in the last paragraph of the Introduction (perhaps after line 91). I especially liked the explanation you provided to Reviewer #2, along the lines of : “Because rats living indoors may function as relatively discreet demographic units (Rahelinirina et al. 2010), we tested the hypothesis that genetic structuring might be more detectable among house-dwelling subpopulations than among outdoor individuals.”

2. Methods: It would benefit your audience to briefly describe 2019-2020 rat control operations in Ankazobe district. Mention if rat control efforts had taken place at your specific study sites before trapping occurred.

3. Methods: Related to point#2, it would also be helpful to mention whether rat removal during your trapping efforts would be expected to significantly reduce rat numbers in residences and surrounding habitat. I am curious to know if rat removal could explain the lower Ne estimates inside houses.

4. Methods: Please add a few more details on the trapping design. Outside the village, you placed 84 traps spaced by 10 m. Were these placed in a single long transect 830m long, or multiple shorter transects? Or in a grid design? If a grid, please give the size of the grid area.

5. Results Line 267: One of the most important results is that genetic structure is higher among rat subpopulations sampled inside houses than among subpopulations sampled outside villages. Please add the two Fst values to this sentence (one for inside houses, one for outside villages) and the range of pairwise Fst values. In fact, it would help readers to see all pairwise Fst values from inside the houses in a table format (probably as a diagonal matrix). A separate diagonal matrix could be used for samples from outside villages. If the authors are willing to do so, these diagonal matrices could be added as supplementary material. This would give a clear understanding of variation among Fst values and show whether the pattern is driven by a few outliers, or is consistent across all pairwise Fst comparisons.

6. Results: Related to point#5 above, is the genetic structure of rats in houses due to relatives remaining in this habitat instead of dispersing? You could answer this by using FSTAT to run a “Comparison among groups of samples” procedure, and choose the Relatedness option to compare samples from inside houses vs. outside villages. This test would provide additional insight into the aspects of rat biology that shape genetic structure at a fine scale.

7. Discussion Line 385: What is meant by “environmental-based management towards reduction of human-rodent contact”? Please give 1 or 2 specific examples. (for instance, do you mean house improvements to prevent holes that allow rat access, or storing food in a shed separate from the living space?)

Minor Revisions: The manuscript is generally well-written, but some revisions are needed for clarity. Here are my suggestions for re-wording specific sentences.

8. Line 22: Change to “In this context, our study investigates…”

9. Line 25: Change to “…and in habitat outside of villages”

10. Line 28: Change to “…relatively similar among villages”

11. Line 36: “Because of the re-colonization problem, an integrated approach…”

12. Line 47: “within or near these natural foci”

13. Line 78: “…which may be associated with the heterogeneous”

14. Line 90: …effective population size (Ne),

15. Line 90: “and examined the short-term stability”

16. Line 132: Remove “purpose”.

17. Line 149: Change to “and manually corrected in GeneMapper…”

18. Line 191: “genetic groups”

19. Line 214: Change human to “humane”

20. Line 237: I suggest replacing homogeneous with “similar” (but this is up to the authors)

21. Line 253-254: Remove the text in this sentence, it repeats the same information as the first sentence. Keep the statistical reporting by adding it to the first sentence.

22. Line 268: Replace “…in one session or in the other” with “…within individual sessions”.

23. Line 272: after Fst = 0.095, add the range of pairwise values.

24. Line 288: use lower case for north-south.

25. Line 326: change “in four areas” to “in four other highland areas”.

26. Line 376-377: Remove the word “although” from the start of the sentence and place it after the first comma “, although with marked seasonal fluctuations, and our results reveal…”.

27. Line 379-381: I recommend merging these two sentences in the following way: At the end of the first sentence add “…fluctuating units, leading to population stability at the district scale. I would then remove the second sentence (Line 380-381) because the term “limited natural turnover” seems confusing in this context.

28. Line 386-387: Consider removing “on the one hand….on the other hand”.

29. Table 1: The order of subpopulations in the table is not the same as in Figure 2 (or Fig. 3). I suggest re-arranging the Table to match the order given in Figure 2 (ABK, AMB, ANK, ANT, KIA, TLA). This will make it much easier for your readers to follow the reporting of results.

30. Fig 1: Increase the size of the inset showing Madgascar, so that readers will be able to find Ankazobe.

31. Fig. 1 caption: Add a period after “district.” Also, end with “…two sessions (2019 and 2020).”

32. Fig 2 caption: Change NE to “Ne”. Remove “s” from subpopulations

33. Fig. 3 caption: add “s” to house. Spell out full name of Rattus rattus for all figure and table captions.

**********

7. PLOS authors have the option to publish the peer review history of their article (what does this mean?). If published, this will include your full peer review and any attached files.

If you choose “no”, your identity will remain anonymous but your review may still be made public.

**Do you want your identity to be public for this peer review?**  For information about this choice, including consent withdrawal, please see our Privacy Policy.

Reviewer #2: No

Reviewer #4: No

Reviewer #5: **Yes:** Gabriele Sgarlata

Reviewer #6: No

**********

[NOTE: If reviewer comments were submitted as an attachment file, they will be attached to this email and accessible via the submission site. Please log into your account, locate the manuscript record, and check for the action link "View Attachments". If this link does not appear, there are no attachment files.]

To ensure your figures meet our technical requirements, please review our figure guidelines: https://journals.plos.org/plosone/s/figures

You may also use PLOS’s free figure tool, NAAS, to help you prepare publication quality figures: https://journals.plos.org/plosone/s/figures#loc-tools-for-figure-preparation.

NAAS will assess whether your figures meet our technical requirements by comparing each figure against our figure specifications.

# Author comment: Author response to Decision Letter 3

**DOI:** 10.1371/journal.pone.0334819.r006

17 Apr 2026

We thank the reviewers for their constructive comments. We carefully addressed all points raised. Detailed responses to each comment are provided below:

Reviewer #2: I have read the revised version of this work and I found that the authors have addressed all my comments satisfactorily. I have no further comments.

Author: We thank the Reviewer #2 for revising this manuscript.

Reviewer #4: The manuscript is clear and well structured, and the objectives, methods, and results are presented in a coherent and understandable manner. The authors have adequately addressed the issues raised during the review process, and the revised version meets the standards for publication. The study contributes relevant data to the understanding of rodent population dynamics in relation to disease surveillance.

I have no additional comments or concerns regarding research ethics or publication ethics. I support the publication of this manuscript.

Author: We thank the Reviewer #4 for his/her time and support.

Reviewer #5: This is my first revision of Parany et al. The study aims at quantifying genetic structure of rat populations occurring inside houses and outside houses across the Ankazobe district in the Central Highlands of Madagascar. This study is relevant in the context of plague outbreak, since rats are one of the major reservoir of plague spread. The authors used 17 microsatellites loci of >400 individuals sampled over two seasons (March-May 2019 and March-May 2020) to investigate genetic diversity, effective population size and genetic structure inside and outside houses, and across sampling seasons. They found higher genetic diversity and effective population size in the outside houses populations compared to the inside houses. There are also evidence of stronger genetic structure in the inside house populations. Overall, they conclude that the higher genetic diversity in the outside populations is likely due to the fact that there is higher dispersal among outside populations than inside populations. Higher dispersal in the outside populations suggest that rodent surveillance plans should not carry out on single subpopulations, but considering the whole network of rat connectivity.

The manuscript is well written and clear. I think that they also present well the context. That being said I am not an expert on disease spread, plague and rat-plague co-dispersal so I cannot judge if the state of the art of the topic has been accurately presented.

I think that the authors have a quite unique dataset, although currently limited to microsatellites. I have some questions and suggestions that may help increase the impact of this work. I suggest the publication of this work upon minor revision.

General Comments

I might have missed it but it would have been interesting to see a quantitative comparison of plague seroprevalence between the inside vs outside houses rat populations. If the authors have enough data to quantify such differences, it would also be interesting to assess whether differences in seroprevalence between inside and outside houses rats correlates with measures of genetic diversity (He and Fst) in the respective populations (inside and outside houses). Also, did the author computed seroprevalence per sampling seasons? Are there difference between those?

Author: Unfortunately, the number of seropositive individuals was very low in our dataset, thus greatly limiting statistical power and precluding robust comparisons between rats from inside versus outside houses: that is the reason why seroprevalence levels were just compared among localities. To make this point clearer, the number of seropositive rodents is now reported in Table 1 of the revised version.

Also, I noticed that the authors reported genetic IBD statistics for the inside houses populations and overall sampling, but not among outside houses rat populations. Did I miss it or there is a specific reason for not doing that?

Author: We agree with the reviewer. Although not significant, IBD statistics are now specified in the revised version for rat subpopulations outside houses (Lines 302- 303).

I think that in this context spatial analyses of genetic structure are quite relevant to understand how rat dispersal impact plague spread. I would suggest the authors to add few other spatial analyses which are related to genetic IBD but different, such as spatial autocorrelation or EEMS analysis (which measure rate of relative dispersal across a landscape using either microsatellite or SNP data) [https://github.com/dipetkov/eems].

Author: We thank the reviewer for this helpful suggestion and agree that spatially explicit analyses such as spatial autocorrelation or EEMS could provide valuable insights into rat dispersal and its potential role in plague spread. Both spatial autocorrelation analyses and EEMS generally require a relatively dense and spatially continuous sampling scheme to reliably infer patterns of gene flow across heterogeneous landscapes. In our study, sampling was limited to six sites located outside villages, which provides limited spatial resolution and does not adequately capture landscape continuity. In addition, our dataset includes 17 microsatellite loci, which, while sufficient for estimating population structure and genetic differentiation, may provide limited precision for spatially explicit inference of migration surfaces, particularly for methods such as EEMS that typically benefit from a larger number of genetic markers (e.g., high-density SNP datasets). For these reasons, we believe that applying these methods to our dataset would not yield robust or biologically interpretable results. Nevertheless, we acknowledge their relevance and agree that such approaches would be valuable in future studies with denser spatial sampling and higher-resolution genomic data.

Detailed Comments (numbering refers to the newest version of the manuscript I received)

Line 296: I think this sentence should be rephrased. The genetic IBD appears to be significant but negative in slope. I see what the authors means, but I think it should be rephrased.

Author: We agree that this sentence is misleading. The reported p-value (p = 0.001) results from a permutation-based test of the overall correlation structure, which can detect very weak statistical associations. However, the estimated slope of the relationship between genetic and geographic distances was extremely small and not significantly different from zero, as indicated by the 95% confidence interval overlapping zero. We therefore interpreted this result as indicating no biologically meaningful isolation by distance. To avoid confusion, we have deleted the reported p-values, in order to make clear that our interpretations were based on slopes and their confidence intervals (lines 301 - 305).

Line 297-299: what about genetic IBD for outside houses populations?

Author: The IBD for rat populations outside houses is not significant. We have now included this result in the revised manuscript (lines 302 - 303).

Line 282: I think it could be nice to better point to the south and northern populations in Fig. 3. I feel it is very difficult to see that at the moment.

Author: We thank the reviewer for this suggestion. However, we share the view of Reviewer #6 that it was probably overstated to interpret the genetic differentiation between ANK and AMB and the other four localities (KIA, ANT, TLA and ABK) as a north–south gradient. The discussion was thus modified on this point in the revised version (see below).

Line 358-360: replace “larger sizes” with “larger effective population size”. Higher genetic diversity or Ne do not directly translate into higher census size, but it might be related to patterns of dispersal, higher in the outside houses than inside houses populations (as also the authors points out in the discussion).

Author: this has been done (line 365).

Line 362-363: Could you measure relative abundance based on rat capture rate over the two sampling seasons and subpopulations?

Author: The results on relative abundances by habitat and by session are fully presented in Parany et al. (2026): these demographic data come from a longitudinal study (2018 – 2020) while the rats genetically analyzed in the present manuscript are only a part of them (2019-2020). Doing so, we were still able to relate the population genetics results to the trends observed in relative abundance across sessions. No significant difference in capture rates was observed between S2019 and S2020 sessions (March – May), indicating stable relative abundance over time. This pattern is consistent with the absence of temporal genetic structure observed in this study.

Reviewer #6: Reviewer's report: Genetic structure of Rattus rattus populations in an endemic plague focus in Madagascar: implications for rodent surveillance and management.

This is my first review of the Parany et al. manuscript and I can see the authors have been thorough in responding to comments of all previous reviewers. This exploratory study provides an interesting comparison of genetic structure among rat subpopulations sampled from two environments: inside houses versus surrounding fields. The authors make key inferences regarding the rapid recolonization of rats, which is significant for plague control efforts in Madagascar.

Overall, the population genetic analyses are robust at both district-wide and local scales. However, some of the inferences are overstated, such as interpreting the genetic differentiation of Ankazobe (ANK) and Ambohitromby (AMB) as being a north-south pattern. ABK (a southern location) does not support a north-south hypothesis, based on the data presented. An alternative explanation that seems more plausible is given in the Discussion; namely, proximity to major urban centers which could potentially lead to gene flow into ANK and AMB. I recommend removing text related to a north-south pattern in the Discussion, and instead focus on the finding that ANK and AMB are members of a genetic group that is distinct from the other four locations.

Author: We agree with the Reviewer for this point. ANK and AMB are localities that are more directly connected with frequent people interactions with other major urban centers such as Antananarivo and Mahajanga than the other four localities (KIA, ANT, TLA and ABK) of the district, and could thus be more likely colonised by rats coming from localities outside the Ankazobe district. We modified the corresponding sentences in Results (Lines –309 - 319) and Discussion sections (Lines 394 - 403).

Major Revisions:

1. Introduction: I saw the main hypothesis described in the Methods on lines 203-206. I think it would make the Introduction stronger if you also mentioned this hypothesis (briefly) in the last paragraph of the Introduction (perhaps after line 91). I especially liked the explanation you provided to Reviewer #2, along the lines of : “Because rats living indoors may function as relatively discreet demographic units (Rahelinirina et al. 2010), we tested the hypothesis that genetic structuring might be more detectable among house-dwelling subpopulations than among outdoor individuals.”

Author: We thank the Reviewer for this suggestion, we have taken it into account and have included this hypothesis in the Introduction in the revised version. Lines 88 – 92.

2. Methods: It would benefit your audience to briefly describe 2019-2020 rat control operations in Ankazobe district. Mention if rat control efforts had taken place at your specific study sites before trapping occurred.

Author: As recalled above, we conducted a longitudinal trapping survey across six localities in Ankazobe, with six trapping sessions. For the genetic analyses, we selected two of these sessions (S.2019 and S.2020). As specified in the revised version, no large-scale rat control operations had been conducted in our study sites prior - to trapping. (Lines 116 – 117).

3. Methods: Related to point#2, it would also be helpful to mention whether rat removal during your trapping efforts would be expected to significantly reduce rat numbers in residences and surrounding habitat. I am curious to know if rat removal could explain the lower Ne estimates inside houses.

Author: While our trapping removed some individuals, it was limited in scope and unlikely to have significantly reduced rat numbers in residences or surrounding habitats. A recent study conducted in Malagasy rural habitats (like those investigated here) using similar protocols showed that trapping inside houses did reduce rodent activity in the very short-term (a few weeks) with no effect on the longer-term, with rodent abundance in similar levels by the following month (Rahelinirina et al., 2021 - Integrative Zoology 2021; 16: 868–885). Therefore, the observed lower Ne estimates inside houses retrieved in our study likely reflect natural population structure rather than an artifact of rat removal.

4. Methods: Please add a few more details on the trapping design. Outside the village, you placed 84 traps spaced by 10 m. Were these placed in a single long transect 830m long, or multiple shorter transects? Or in a grid design? If a grid, please give the size of the grid area.

Author: We placed the 84 traps spaced by 10 m in a single long transect 830m long. We have added this detail in the Lines 133-137.

5. Results Line 267: One of the most important results is that genetic structure is higher among rat subpopulations sampled inside houses than among subpopulations sampled outside villages. Please add the two Fst values to this sentence (one for inside houses, one for outside villages) and the range of pairwise Fst values. In fact, it would help readers to see all pairwise Fst values from inside the houses in a table format (probably as a diagonal matrix). A separate diagonal matrix could be used for samples from outside villages. If the authors are willing to do so, these diagonal matrices could be added as supplementary material. This would give a clear understanding of variation among Fst values and show whether the pattern is driven by a few outliers, or is consistent across all pairwise Fst comparisons.

Author: Fst values for inside houses and outside villages subpopulations as well as the range of pairwise Fst values have been added in lines 290 - 294. Furthermore, we provided all pairwise Fst values from both inside the houses and outside villages as diagonal matrices in the S2 Supplementary Table (lines 649 – 653). Only one pairwise FST value was not significant for pairs of subpopulations sampled inside houses, compared to ten values for pairs of subpopulations sampled outside villages (see Table S2). Moreover, a boxplot graph illustrates that the pattern of higher genetic structure difference among rat subpopulations sampled inside houses than among subpopulations sampled outside villages is not driven by a few outliers.

6. Results: Related to point#5 above, is the genetic structure of rats in houses due to relatives remaining in this habitat instead of dispersing? You could answer this by using FSTAT to run a “Comparison among groups of samples” procedure, and choose the Relatedness option to compare samples from inside houses vs. outside villages. This test would provide additional insight into the aspects of rat biology that shape genetic structure at a fine scale.

Author: We thank the Reviewer for this suggestion. We compared relatedness within subpopulations sampled inside houses and outside villages using FSTAT, and found higher levels within subpopulations sampled inside houses, as it is expected under the hypothesis of low dispersal. This analysis has now been included in the Methods, Results and Discussions sections (Lines 181 – 182; 257 – 261; 388 - 390).

7. Discussion Line 385: What is meant by “environmental-based management towards reduction of human-rodent contact”? Please give 1 or 2 specific examples. (for instance, do you mean house improvements to prevent holes that allow rat access, or storing food in a shed separate from the living space?)

Author: We have added some examples in the Discussion section to clarify “environmental-based management towards reduction of human-rodent contact,” Lines 421 - 422.

Minor Revisions: The manuscript is generally well-written, but some

Attachment Submitted filename: Response to reviewer_3_Parany.docx

# Peer review: Decision Letter 3

**DOI:** 10.1371/journal.pone.0334819.r007

**Authors:** Tzen-Yuh Chiang, Tzen-Yuh Chiang, Tzen-Yuh Chiang, Tzen-Yuh Chiang

13 May 2026

Genetic structure of *Rattus rattus*  populations in an endemic plague focus in Madagascar: implications for rodent surveillance and management

PONE-D-25-53545R3

Dear Dr. Carine Brouat,

We’re pleased to inform you that your manuscript has been judged scientifically suitable for publication and will be formally accepted for publication once it meets all outstanding technical requirements.

Within one week, you’ll receive an e-mail detailing the required amendments. When these have been addressed, you’ll receive a formal acceptance letter and your manuscript will be scheduled for publication.

An invoice will be generated when your article is formally accepted. Please note, if your institution has a publishing partnership with PLOS and your article meets the relevant criteria, all or part of your publication costs will be covered. Please make sure your user information is up-to-date by logging into Editorial Manager at Editorial Manager® and clicking the ‘Update My Information' link at the top of the page. For questions related to billing, please contact billing support.

If your institution or institutions have a press office, please notify them about your upcoming paper to help maximize its impact. If they’ll be preparing press materials, please inform our press team as soon as possible -- no later than 48 hours after receiving the formal acceptance. Your manuscript will remain under strict press embargo until 2 pm Eastern Time on the date of publication. For more information, please contact onepress@plos.org.

Kind regards,

Tzen-Yuh Chiang

Academic Editor

PLOS One

Additional Editor Comments (optional):

Reviewers' comments:

Reviewer's Responses to Questions

**Comments to the Author**

1. If the authors have adequately addressed your comments raised in a previous round of review and you feel that this manuscript is now acceptable for publication, you may indicate that here to bypass the “Comments to the Author” section, enter your conflict of interest statement in the “Confidential to Editor” section, and submit your "Accept" recommendation.

Reviewer #2: (No Response)

Reviewer #5: All comments have been addressed

Reviewer #6: All comments have been addressed

**********

2. Is the manuscript technically sound, and do the data support the conclusions?

The manuscript must describe a technically sound piece of scientific research with data that supports the conclusions. Experiments must have been conducted rigorously, with appropriate controls, replication, and sample sizes. The conclusions must be drawn appropriately based on the data presented.

Reviewer #2: (No Response)

Reviewer #5: Yes

Reviewer #6: Yes

**********

3. Has the statistical analysis been performed appropriately and rigorously?

Reviewer #2: (No Response)

Reviewer #5: Yes

Reviewer #6: Yes

**********

4. Have the authors made all data underlying the findings in their manuscript fully available?

The PLOS Data policy requires authors to make all data underlying the findings described in their manuscript fully available without restriction, with rare exception (please refer to the Data Availability Statement in the manuscript PDF file). The data should be provided as part of the manuscript or its supporting information, or deposited to a public repository. For example, in addition to summary statistics, the data points behind means, medians and variance measures should be available. If there are restrictions on publicly sharing data—e.g. participant privacy or use of data from a third party—those must be specified.

Reviewer #2: (No Response)

Reviewer #5: Yes

Reviewer #6: Yes

**********

5. Is the manuscript presented in an intelligible fashion and written in standard English?

PLOS ONE does not copyedit accepted manuscripts, so the language in submitted articles must be clear, correct, and unambiguous. Any typographical or grammatical errors should be corrected at revision, so please note any specific errors here.

Reviewer #2: (No Response)

Reviewer #5: Yes

Reviewer #6: Yes

**********

6. Review Comments to the Author

Please use the space provided to explain your answers to the questions above. You may also include additional comments for the author, including concerns about dual publication, research ethics, or publication ethics. (Please upload your review as an attachment if it exceeds 20,000 characters)

Reviewer #2: I found that the authors have made a substantial effort to adequately respond to all reviewers comments including myself. I have no further comments and I support the acceptance of the manuscript.

Reviewer #5: The authors have carefully replied to my comments. Therefore, I support the publication of the manuscript.

Reviewer #6: The authors have made extensive edits that have significantly improved the manuscript. I appreciate their mindful consideration of comments from all reviewers. I have no remaining concerns and only a few final edits to suggest.

Minor edits:

1. Line 114: In Fig. 1 legend, please capitalize “March”

2. Line 137: Change to “one kidney from each….”

3. Line 168: Please consider revising this sentence to “Genetic diversity of each subpopulation was estimated with FSTAT v2.9.4 using three estimators: 1) allelic richness..., 2) Nei’s ..., and 3) the inbreeding coefficient...”. This will make it easier for your readers to understand.

4. Line 170: Nei should not be italicized

5. Line 248: Remove “relatedness” because it is redundant. You already mentioned this term in the first word of the sentence

6. Line 252: Table 1 title, remove “s” from subpopulations

7. Line 356: Remove “of”

8. Line 366: Change “observed within” to “of the”

9. Line 375: Change “with frequent people interactions” to “frequent human movements”

**********

7. PLOS authors have the option to publish the peer review history of their article (what does this mean?). If published, this will include your full peer review and any attached files.

If you choose “no”, your identity will remain anonymous but your review may still be made public.

**Do you want your identity to be public for this peer review?**  For information about this choice, including consent withdrawal, please see our Privacy Policy.

Reviewer #2: No

Reviewer #5: **Yes:** Gabriele Sgarlata

Reviewer #6: No

**********

# Editor report: Acceptance letter

**DOI:** 10.1371/journal.pone.0334819.r008

**Authors:** Tzen-Yuh Chiang, Tzen-Yuh Chiang, Tzen-Yuh Chiang, Tzen-Yuh Chiang

PONE-D-25-53545R3

PLOS One

Dear Dr. Brouat,

I'm pleased to inform you that your manuscript has been deemed suitable for publication in PLOS One. Congratulations! Your manuscript is now being handed over to our production team.

At this stage, our production department will prepare your paper for publication. This includes ensuring the following:

* All references, tables, and figures are properly cited

* All relevant supporting information is included in the manuscript submission,

* There are no issues that prevent the paper from being properly typeset

You will receive further instructions from the production team, including instructions on how to review your proof when it is ready. Please keep in mind that we are working through a large volume of accepted articles, so please give us a few days to review your paper and let you know the next and final steps.

Lastly, if your institution or institutions have a press office, please let them know about your upcoming paper now to help maximize its impact. If they'll be preparing press materials, please inform our press team within the next 48 hours. Your manuscript will remain under strict press embargo until 2 pm Eastern Time on the date of publication. For more information, please contact onepress@plos.org.

You will receive an invoice from PLOS for your publication fee after your manuscript has reached the completed accept phase. If you receive an email requesting payment before acceptance or for any other service, this may be a phishing scheme. Learn how to identify phishing emails and protect your accounts at https://explore.plos.org/phishing.

If we can help with anything else, please email us at customercare@plos.org.

Thank you for submitting your work to PLOS ONE and supporting open access.

Kind regards,

PLOS ONE Editorial Office Staff

on behalf of

Dr. Tzen-Yuh Chiang

Academic Editor

PLOS One
